# Genomic prediction and QTL analysis for grain Zn content and yield in *Aus*-derived rice populations

**DOI:** 10.1007/s13562-024-00886-0

**Published:** 2024-05-09

**Authors:** Tapas Kumer Hore, C. H. Balachiranjeevi, Mary Ann Inabangan-Asilo, C. A. Deepak, Alvin D. Palanog, Jose E. Hernandez, Glenn B. Gregorio, Teresita U. Dalisay, Maria Genaleen Q. Diaz, Roberto Fritsche Neto, Md. Abdul Kader, Partha Sarathi Biswas, B. P. Mallikarjuna Swamy

**Affiliations:** 1 International Rice Research Institute (IRRI), DAPO Box 4031, Los Banos, Laguna Philippines; 2 University of the Philippines Los Baños (UPLB), College, Los Banos, Laguna Philippines; 3 Bangladesh Rice Research Institute (BRRI), Gazipur, Bangladesh; 4 University of Agricultural Sciences, Bangalore, Karnataka India; 5 Southeast Asian Regional Center for Graduate Study and Research in Agriculture (SEARCA), Los Banos, Philippines; 6 LSU Ag Center, Louisiana State University, Baton Rouge, LA USA

**Keywords:** Rice, RIL, Zn, Yield, GWAS, QTL, Genes

## Abstract

Zinc (Zn) biofortification of rice can address Zn malnutrition in Asia. Identification and introgression of QTLs for grain Zn content and yield (YLD) can improve the efficiency of rice Zn biofortification. In four rice populations we detected 56 QTLs for seven traits by inclusive composite interval mapping (ICIM), and 16 QTLs for two traits (YLD and Zn) by association mapping. The phenotypic variance (PV) varied from 4.5% (*qPN*_*4.1*_) to 31.7% (*qPH*_*1.1*_). *qDF*_*1.1*_, *qDF*_*7.2*_, *qDF*_*8.1*_, *qPH*_*1.1*_, *qPH*_*7.1*_, *qPL*_*1.2*_, *qPL*_*9.1,*_* qZn*_*5.1*_, *qZn*_*5.2*_, *qZn*_*6.1*_ and *qZn*_*7.1*_ were identified in both dry and wet seasons; *qZn*_*5.1*_*, qZn*_*5.2*_, *qZn*_*5.3,*_* qZn*_*6.2,*_* qZn*_*7.1*_ and *qYLD*_*1.2*_ were detected by both ICIM and association mapping. *qZn*_*7.1*_ had the highest PV (17.8%) and additive effect (2.5 ppm). Epistasis and QTL co-locations were also observed for different traits. The multi-trait genomic prediction values were 0.24 and 0.16 for YLD and Zn respectively. *qZn*_*6.2*_ was co-located with a gene (*OsHMA2*) involved in Zn transport. These results are useful for Zn biofortificatiton of rice.

**Supplementary Information:**

The online version contains supplementary material available at 10.1007/s13562-024-00886-0.

## Introduction

Zinc (Zn) is critical for human health due to its vital role in diverse metabolic processes, catalytic activities, and highly essential for the functioning of numerous proteins (Aggett 2020; Thompson 2022). Zn deficiency causes stunting, diarrhea, reduced immunity, poor growth and development, and increased vulnerability to diabetes and COVID-19. Severe Zn deficiency is even linked to maternal and child mortality (Aggett 2020; Farooq et al. 2020; Hussein et al. 2021; Peramaiyan et al. 2022; Ryu and Aydemir 2020; Shahzad et al. 2014; Vogel-González et al. 2021). Particularly prevalent in impoverished and marginalized populations relying solely on staple cereals for their nutritional needs, the lack of resources, access to a diverse diet, supplementation, or fortified foods exacerbates Zn deficiency-related health issues (Semba et al. 2022). With estimates indicating that over half of the global population faces micronutrient deficiency (Palanog et al. 2019; Shahzad et al. 2014), addressing health and nutrition of children, women, and vulnerable populations has become a pressing global health priority aligned with sustainable development goals (Global Nutrition Report 2021).

Biofortification of staple food crops has been taken up to tackle nutritional deficiencies in the developing world (Bouis and Saltzman 2017; Pradhan et al.^2020^). Rice is biofortified with Fe, Zn and Vitamin A, and other important mineral elements (Calayugan et al. 2021; Kader et al. 2020; Swamy et al. 2021). However, increasing the grain Zn content of the popular rice varieties by 6–12 ppm in the endosperm has been the major focus; these varieties can meet up to 30–40% of the estimated daily average requirement of Zn, which is enough to overcome most of the Zn deficiency-related health problems in the target populations (Pradhan et al. 2020; Swamy et al. 2016). The initial success, especially in South Asia, South East Asia, and Africa (Kader et al. 2021; Palanog et al. 2019), has spurred demand for Zn-enriched rice varieties in other target countries and paved the way for mainstream Zn breeding to make grain Zn an integral trait in all future rice varieties (Bouis and Saltzman 2017). Urgently required in this pursuit is the identification and molecular characterization of novel high-Zn rice germplasms that are genetically close to elite breeding lines, facilitating their efficient integration into genomics-assisted Zn mainstream breeding (Calayugan et al. 2021).

The grain Zn trait in rice has been extensively characterized phenotypically and molecularly (Babu et al. 2020; Calayugan et al. 2020; Descalsota et al. 2018, 2019a; Zaw et al. 2019), revealing over 200 QTLs and 80 genes associated with grain Zn content (De Leon et al. 2016; Furuta et al. 2017; Singhal et al. 2021; Swamy et al. 2016; Yang et al. 2018). Meta-analyses have further refined this knowledge, identifying 22 to 57 Meta QTLs (MQTLs) enriched with metal homeostasis genes crucial for Zn uptake, transport, partition, and loading into rice grains (Jin et al. 2015; Raza et al. 2019;Joshi et al. 2023; Sasaki et al. 2014, 2015; Satoh-Nagasawa et al. 2012; Takahashi et al. 2012a; Tan et al. 2019). The complexity of plant nutrition mechanisms, influenced strongly by environmental factors, is evident in the differential expression, co-expression, and transgenic validation of major metal homeostasis genes such as *OsDMAS, OsHMA*, *OsMTP, OsNAS, OsNAAT, OsSAMS, OsTOM,* and *OsZIP* (Johnson et al. 2011; Malav et al. 2016; Mu et al. 2021; Sasaki et al. 2015; Satoh-Nagasawa et al. 2012; Takahashi et al. 2012b; Tan et al. 2019; Yamaji et al. 2013; Descalsota et al. 2019a, b; Zaw et al. 2019).

The “*aus”* accessions are a rich source of grain micronutrients, especially for Zn content in the milled rice, and they are genetically closer to popular *indica* rice varieties so that they can be readily used in rice Zn biofortification (Descalsota et al. 2018; Rakotondramanana et al. 2022; Swamy et al. 2016). Some of the stable high Zn *aus* accessions are Kaliboro (IRGC77201-1), Jamir (IRGC117765), Lalsaita (IRGC43915-1), UCP122 (IRGC8794-1), DZ193, and Khao ToT Long 227. In addition to their acceptable yield potential, these accessions have two to three folds higher grain Zn content (35–40 ppm) compared to the popular high yielding rice varieties (12–16 ppm). Our team at International Rice Research Institute (IRRI) and our research collaborators are using these accessions for Zn biofortification breeding. Previous mapping studies using *aus*-derived populations have identified major effect QTLs such as *Zn*_*5.1*_, *Zn*_*6.1*_, *Zn*_*6.2*_, *Zn*_*7.1*_, and *Zn*_*12.1*_ with substantial R^2^ values (> 10%) and additive effect (> 1 ppm) (Descalsota et al. 2019a,b; Zaw et al. 2019), emphasizing their potential to enhance grain Zn content through genomics-assisted breeding (Neeraja et al. 2017; Swamy et al. 2016; Wani et al. 2022).

In support of mainstream Zn breeding, our study aims to characterize four recombinant inbred lines (RILs) populations for agronomic, yield, and grain Zn content traits; to map quantitative trait loci (QTLs) and assess the pyramiding effects of yield and Zn QTLs; to prioritize candidate genes for novel QTLs; to conduct genomic prediction for grain Zn and yield; and to identify promising RILs with high grain Zn content and acceptable yield potential.

## Materials and methods

### Plant materials

Four RILs mapping populations were developed and characterized in this study. Two popular irrigated rice varieties of the Philippines such as PSBRc82 and NSICRc222 were crossed with high Zn *aus* accession UCP122 (IRGC8794-1), while two submergence tolerant rice varieties viz; Samba Mahsuri Sub-1 and Ciherang Sub-1 were crossed with another *aus* accession Lalsaita (IRGC43915-1). We followed all the procedures and methods as per the established regulations and guidelines.

### Phenotyping

Four populations viz; PSBRc82/UCP122 (P1), NSICRc222/UCP122 (P2), Samba Mahsuri Sub-1/Lalsaita (P3) and Ciherang Sub-1/Lalsaita (P4) along with checks were field evaluated during the wet season of 2019 (2019WS) and dry season of 2020 (2020DS). Experiments were conducted at Zeigler Experimental Station (ZES) of IRRI. Twenty-one days old seedlings were transplanted with a spacing of 20 cm × 20 cm following a Randomized Complete Block Design (RCBD) with two replications. Data were recorded on days to flowering (DF), plant height (PH), tiller number (TN), panicle number (PN), panicle length (PL), grain yield (YLD), and grain Zn content (Zn) following the standard evaluation system (IRRI 2013). For yield measurement, grains were threshed per plot and dried to 14% moisture content and yield was recorded in grams (g) and converted to tons/ha. Thirty grams of paddy was dehulled using Satake dehuller and milled for 60 s using mini-lab rice polisher from green agritech. At least 3 g of representative polished rice grains from each plot was analyzed for Zn content using X-ray Fluorescence Spectrometry (XRF) (Guild et al. 2017). Each sample was measured twice to estimate grain Zn content and expressed in parts per million (ppm). The replicated data was used for statistical analysis.

### Molecular analysis

#### DNA isolation and quantification

Leaf samples of all the RILs were collected from one month old seedlings for all the genotypes from a single replication. Genomic DNA was isolated following the Cetyl-Trimethyl Ammonium Bromide (CTAB) method (Murray and Thompson 1980). The lyophilized leaf samples were ground into powder using liquid Nitrogen and GenoGrinder (SPEX sample prep 2000). Pre-warmed 800 µl CTAB buffer was added to each of the samples and incubated at 65 °C for 1 h, added 800 µl of chloroform iso-amyl alcohol (1:1) and mixed very well by shaking followed by centrifugation for 15 min at 12,000 revolutions per minute (rpm). The supernatant was carefully transferred to a new tube and an equal volume of chilled isopropyl alcohol was added and incubated for 30 min at 4 °C. Next, the DNA was centrifuged at 10,000 rpm for 10 min and the pellet was washed with 70% alcohol and air dried. Finally, the DNA pellet was dissolved in 200 µl of TE buffer (10 mM Tris–HCl [pH 8.0], 1 mM EDTA, RNase A [10 mg/ml]). The quality and quantity of the isolated genomic DNA was estimated through 1% agarose gel electrophoresis (AGE), and the final concentration of the DNA samples were adjusted to 25–50 ng/µl. The 7 K SNP genotyping was carried out at Genotyping Sequence Laboratory, IRRI (Morales et al. 2020).

### Statistical analysis

#### Analyses of statistical paremeters and ANOVA

The basic statistical parameters and analysis of variance (ANOVA) were estimated using STAR v.2.0.1 and PB Tools v.1.4 (IRRI 2014). Best linear unbiased estimates (BLUEs) were generated by setting genotype effects as fixed and season effects as random. The BLUEs were used for further analyses. Pearson’s correlation coefficient and Principal Component Analysis (PCA) were performed using R-Program (R core Software 2012).

The model used for ANOVA:

$${Y}_{ijk}= \mu + {\alpha }_{i}+{r}_{j}+{b}_{kj}+ {\varepsilon }_{ijk}$$

where* μ* is the overall mean, *α*_*i*_ is the effect of the *ith* genotype; *r*_*j*_ is the effect of the *jth* replicate, *b*_*kj*_ the effect of the *kth* block within the *jth* replicate and *ε*_*ijk*_ the error.

Broad-sense heritability (h^2^) was estimated by using the formula:

$${h}^{2}=\frac{{\sigma }_{g}^{2}}{{\sigma }_{p}^{2}} and {\sigma }_{p}^{2}= {\sigma }_{g}^{2}+\frac{{\sigma }_{e}^{2}}{r}$$

where ${\sigma }_{p}^{2}$ is the phenotypic variance, ${\sigma }_{g}^{2}$ is the genotypic variance, ${\sigma }_{e}^{2}$ is the error variance and r is the number of replications in the season.

#### QTL analysis by inclusive composite interval mapping

High quality SNPs were retrieved after filtering out for no calls, monomorphic and heterozygous based on parental genotypic information from the 7 K SNP genotyping. In all 848, 211, 398, and 784 SNPs in P1-P4 respectively were used for further analyses. We constructed the linkage map following the Kosambi function and using IciMapping (IciM) v4.2 (Kosambi 1944; Meng et al. 2015). The BLUEs and SNPs data were used in composite interval QTL mapping. The permutation method obtained an empirical threshold for claiming QTLs based on maximum likelihood with 1000 permutations at *P* = *0.05* (Li et al. 2007)*.* The epistasis between QTLs were detected using the maximum likelihood permutation method with 1000 permutation at *P* = *0.05* (Li et al. 2008).

#### Association mapping

The 7 K SNPs data was curated by removing missing data, minor alleles and heterozygous loci using Tassel v.5.0 (Bradbury et al. 2007). In all 2005 SNPs across the populations were retrieved and used for GWAS analysis. GWAS was performed using the Compressed Mixed Linear Model (CMLM) in GAPIT v3 (Zhang et al. 2010). Manhattan plots were obtained by setting a threshold value for declaring marker-trait association at –log (*p*-value) ≥ 2.6. The QQ plots for each trait were carefully examined to determine the population stratification and whether the model could control false positives, which, in turn, indicated the suitability of the model for analysis (Kaler et al. 2020).

#### Estimation of QTL effects

The genotypes were classified based on the number of mapped QTLs for grain Zn and YLD. Each QTL group's average grain Zn and YLD were calculated. Tukeys’s Honest Significant Difference (HSD) analysis was applied to differentiate the QTL classes.

### Candidate gene analysis

In-silico post QTL analysis was performed based on web based available information by using RAP-DB (https://rapdb.dna.affrc.go.jp/) (accessed on 15 April 2022), MSU Rice genome annotation project (http://rice.uga.edu/) and RiceXpro (https://ricexpro.dna.affrc.go.jp/) (accessed on 19 June 2022) to predict responsible candidate genes within the novel QTL interval region. All the candidate genes observed between the two flanking markers of the novel QTL were downloaded and characterized based on expressed proteins and their functions. In addition, in-silico candidate gene expression analysis was also performed through RiceXpro (https://ricexpro.dna.affrc.go.jp/) to explore the information regarding predicted candidate gene expression in different plant parts at different stages and their association with grain Zn. In-silico co-expression analyses was carried out for selected genes using online resource (https://ricefrend.dna.affrc.go.jp/).

### Genomic prediction ability

Genomic prediction was estimated through single-trait (YLD/Zn individually) and multi-trait (YLD and Zn combined) strategies for all populations (Lyra et al. 2017). For that, we ran the linear mixed models via ASReml-R (Analysis Software for Residual Maximum Likelihood following a CV-alpha) cross-validation system with four replicates and 5-folds (Yassue et al. 2021). As the response variable, we used deregressed BLUPs (dBLUPS) for each trait. Moreover, the genotype effect was considered random, and season as fixed in the GS (Genomic Selection) model. Finally, for each replicate and fold, we estimated prediction ability (Pearson correlation between the predicted and observed value) and heritability (broad sense) through the Cullis method (Cullis et al. 2006; Piepho and Möhring 2007).

## Results

### Phenotypic analysis of the four RILs populations

The range, mean, variance, coefficient of variation (CV) and heritability for agronomic traits, grain yield and grain Zn content are provided in Table 1. Days to flowering (DF) ranged from 71–109 days and 60–110 days irrespective of the populations in season 1 (S1) and season 2 (S2), respectively. While the highest values for plant height (PH) (192 cm) and panicle length (PL) (38 cm) were recorded in P1 and P2, respectively. Similarly, the highest values for panicle number (PN) (32) and yield (YLD) (8.9 t/ha) were observed in P3, while tiller number (TN) (27) and grain Zinc content (Zn) (38.1 ppm) were highest in P4. Across the seasons and populations, yield and Zn ranged from 1.5–8.9 t/ha and 10.0–38.1 ppm respectively, whereas in the parents, YLD and Zn ranged from 1.5–5.4 t/ha and 10.2–34.4 ppm, respectively (Table 1). The CV was low (⪅ 10%) for DF and PL, high CV was observed for YLD (> 20%) and rest of the traits had a moderate CV (⪅ 20). Genotypic effects were significant for all the traits and in both seasons. Moderate to high heritability (0.40–0.89) was observed for all traits except TN.

**Table 1:** Performance and heritability of agronomic traits, yield and grain Zn content in RILs over two seasons and across populations

Trait	Pop	RP (Mean)	DP (Mean)	Range		Mean ± SE		Variance		CV(%)		F-Value		H^2^		Combined analysis		
				S1	S2	S1	S2	S1	S2	S1	S2	S1	S2	S1	S2	Geno effect	Seasonal effect	G × E effect
DF	P1	99	77	71–106	71–110	86.2 ± 0.27	86.1 ± 0.28	43.62	41.99	7.66	7.53	7.08***	5.54***	0.85	0.81	15.52**	0.00ns	0.00ns
	P2	98	77	71–109	73–100	87.3 ± 0.28	86.1 ± 0.27	37.29	31.83	7.00	6.55	5.24***	3.58***	0.79	0.71	2.39***	0.12ns	69.58*
	P3	86	73	71–105	64–91	85.2 ± 0.27	81.5 ± 0.15	39.33	12.23	7.35	4.29	5.84***	3.81***	0.82	0.70	1.58***	4.84*	174.69*
	P4	79	75	71–104	60–93	85.6 ± 0.29	81.3 ± 0.23	39.71	24.17	7.36	6.05	5.57***	4.53***	0.82	0.76	2.79***	4.71*	74.28***
PH	P1	102	128	83–190	88–192	136.9 ± 0.85	138.1 ± 0.96	413.5	512.87	14.85	16.4	5.59***	4.39***	0.83	0.77	11.03**	0.15ns	0.00ns
	P2	99	127	101–181	89–187	144.1 ± 0.85	143.8 ± 0.95	322.07	406.15	12.44	14.01	10.54***	4.71***	0.87	0.78	657.98***	0.00ns	0.00ns
	P3	108	129	88–183	89–183	138.1 ± 0.75	138.2 ± 0.75	309.99	308.91	12.75	12.72	7.44***	4.53***	0.86	0.77	1.35***	0.00ns	0.00ns
	P4	117	127	87–175	72–191	133.4 ± 0.85	133.6 ± 0.89	332.79	371.63	13.67	14.43	8.12***	5.80***	0.88	0.82	19.61***	0.00ns	0.00ns
TN	P1	15	18	8–21	7–22	13.3 ± 0.10	12.4 ± 0.13	5.66	5.29	17.89	18.22	1.50***	1.17**	0.43	0.34	2.85***	4.09*	0.00ns
	P2	14	16	8–23	7–21	13.1 ± 0.17	13.1 ± 0.12	13.56	6.72	28.09	19.92	1.02**	1.14*	0.48	0.34	2.09***	3.45*	0.00ns
	P3	14	13	7–24	8–24	12.7 ± 0.10	12.2 ± 0.12	5.42	6.68	18.38	10.07	1.37***	1.51***	0.47	0.34	4.36***	0.00ns	0.00ns
	P4	15	15	7–21	7–27	12.4 ± 0.11	12.6 ± 0.13	5.57	7.66	18.94	21.92	1.27**	1.11*	0.51	0.41	0.92ns	0.00ns	0.04ns
PN	P1	12	13	6–22	6–23	12.4 ± 0.10	14.8 ± 0.08	6.19	3.68	20.05	12.92	1.17**	1.65**	0.55	0.43	11.65**	0.00ns	0.00ns
	P2	12	13	6–20	7–26	12.1 ± 0.11	13.3 ± 0.10	5.91	6.03	20.01	18.19	1.30**	1.45**	0.45	0.39	7.89*	3.00*	0.46ns
	P3	12	12	6–32	7–21	12.0 ± 0.11	12.6 ± 0.10	6.59	5.29	21.46	18.22	1.31***	1.23*	0.44	0.43	5.32*	0.02ns	0.00ns
	P4	13	12	5–20	6–23	11.7 ± 0.11	12.6 ± = 0.11	5.92	2.18	20.84	12.42	1.06**	1.17**	0.47	0.48	4.57*	0.19ns	0.09ns
PL	P1	24.0	23.1	20–34	16–36	26.6 ± 0.11	27.1 ± 0.15	7.29	12.02	10.17	12.81	2.70***	1.99***	0.63	0.50	4.58**	3.75*	0.00ns
	P2	23.9	23.7	20–37	19–38	27.4 ± 0.13	27.3 ± 0.14	8.16	8.81	10.42	10.86	2.29***	2.25***	0.55	0.55	6.71**	0.00ns	0.00ns
	P3	22.8	21.1	19–33	19–34	25.8 ± 0.10	25.8 ± 0.10	5.71	5.77	9.27	9.30	2.42***	2.68***	0.57	0.62	7.68***	0.00ns	0.00ns
	P4	22.7	21.8	18–33	17–32	25.6 ± 0.12	25.6 ± 0.13	6.68	7.29	10.07	10.53	2.00***	1.86***	0.48	0.45	5.62***	0.00ns	0.00ns
YLD	P1	3.8	3.1	1.5–7.1	1.6–8.2	4.1 ± 0.05	4.1 ± 0.05	1.6	1.25	31.55	27.29	2.49***	3.19***	0.60	0.68	1.13*	0.00ns	79.98***
	P2	5.4	3.1	1.9–8.4	1.7–8.4	4.4 ± 0.06	4.4 ± 0.05	1.68	1.3	29.27	25.82	1.27***	3.73***	0.62	0.73	1.45**	0.00ns	16.89***
	P3	4.3	1.5	1.5–8.9	1.7–8.2	4.2 ± 0.06	4.7 ± 0.06	1.72	1.78	31.67	28.13	1.94***	2.80***	0.46	0.63	1.41**	1.73*	37.23***
	P4	3.2	1.9	1.5–8.1	1.8–8.3	4.0 ± 0.06	4.9 ± 0.06	1.51	1.46	30.48	24.49	2.01***	1.54***	0.48	0.42	1.35***	4.06*	11.17***
Zn	P1	10.8	34.4	10.8–35.8	10.3–35.3	22.5 ± 0.17	21.5 ± 0.15	16.88	12.14	18.26	16.24	4.54***	3.38***	0.77	0.68	5.79***	0.16ns	0.00ns
	P2	10.2	31.7	10.2–35.0	10.5–31.7	21.0 ± 0.19	20.5 ± 0.15	16.18	11.17	19.18	16.32	4.11***	4.31***	0.74	0.76	5.95***	0.21ns	0.11ns
	P3	11.1	27.6	10.0–33.7	10.5–35.2	21.7 ± 0.16	22.4 ± 0.15	14.77	13.2	17.7	16.21	3.58***	9.45***	0.72	0.89	5.29***	0.52ns	0.07ns
	P4	10.8	29.6	10.6–38.1	10.3–30.9	20.7 ± 0.15	20.8 ± 0.14	10.63	9.5	15.75	14.83	3.03***	3.95***	0.61	0.73	4.29***	0.00ns	0.01ns

Pop-population; 2019WS, S2-2021DS, SE, standard error; CV, coefficient of variation; H^2^, heritability; Geno effect, genotypic effect; G × E effect, genotype by seasonal effect; DF, days to flowering (days); PH, plant height (cm); TN, tiller number; PN, panicle number; PL, panicle length (cm); YLD, yield (ton/ha); Zn, zinc (ppm); RP, recipient parents; DP, donor parents; ***Significant at *P* < 0.0001; **Significant at *P* < 0.01; *Significant at *P* < 0.05; ns, non-significant (color figure online)

**Fig. 1:**
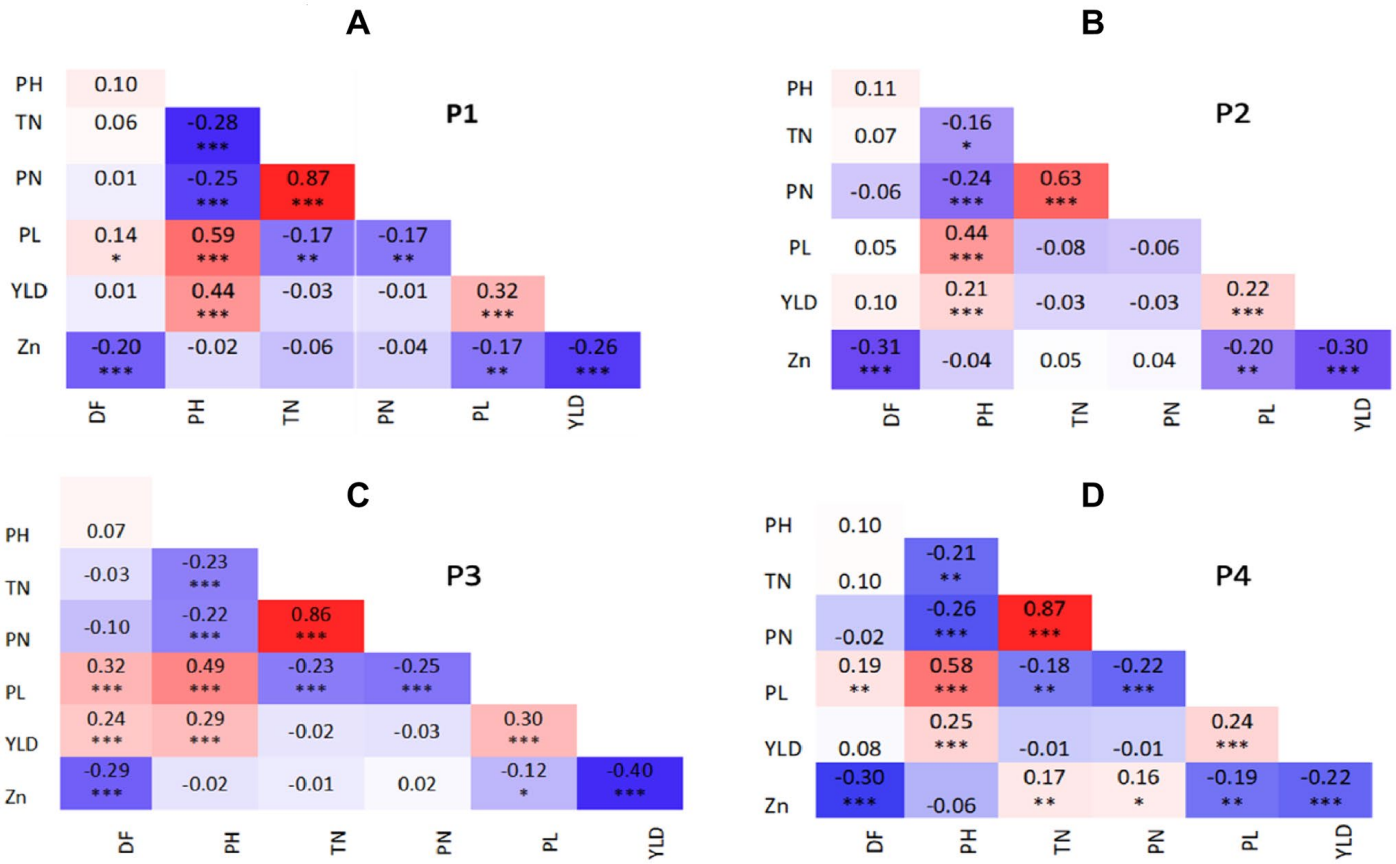
Correlations among agronomic traits, yield, and grain Zn in four RIL populations. Corrleation for each population was estimated using R-Program. Postitive correlations are indicated by red color, while negative correlations are indicated by blue color. Darker colors correspond to stronger correlation coefficients. **A**–**D** indicates correlations for four populations P1–P4; DF, days to flowering (days); PH, plant height (cm); TN, tiller number; PN, panicle number; PL, panicle length (cm); YLD, yield (ton/ha); Zn, zinc (ppm). *p < 0.05, **p < 0.01, ***p < 0.001 (color figure online)

Out of 84 possible pairwise correlations, 47 were significant, 22 of them had positive and 25 had negative correlations (Fig. 1). Positive correlations were observed between PH and PL in all the populations. In contrast, YLD showed a consistent negative association with Zn. We observed significant positive (PN–TN, PL–PH, PL–YLD, PH–YLD) and negative (TN–PH, PN–PH, PL–TN, PL–PN, PL–Zn, YLD–Zn) correlations among the traits.

**Fig. 2:**
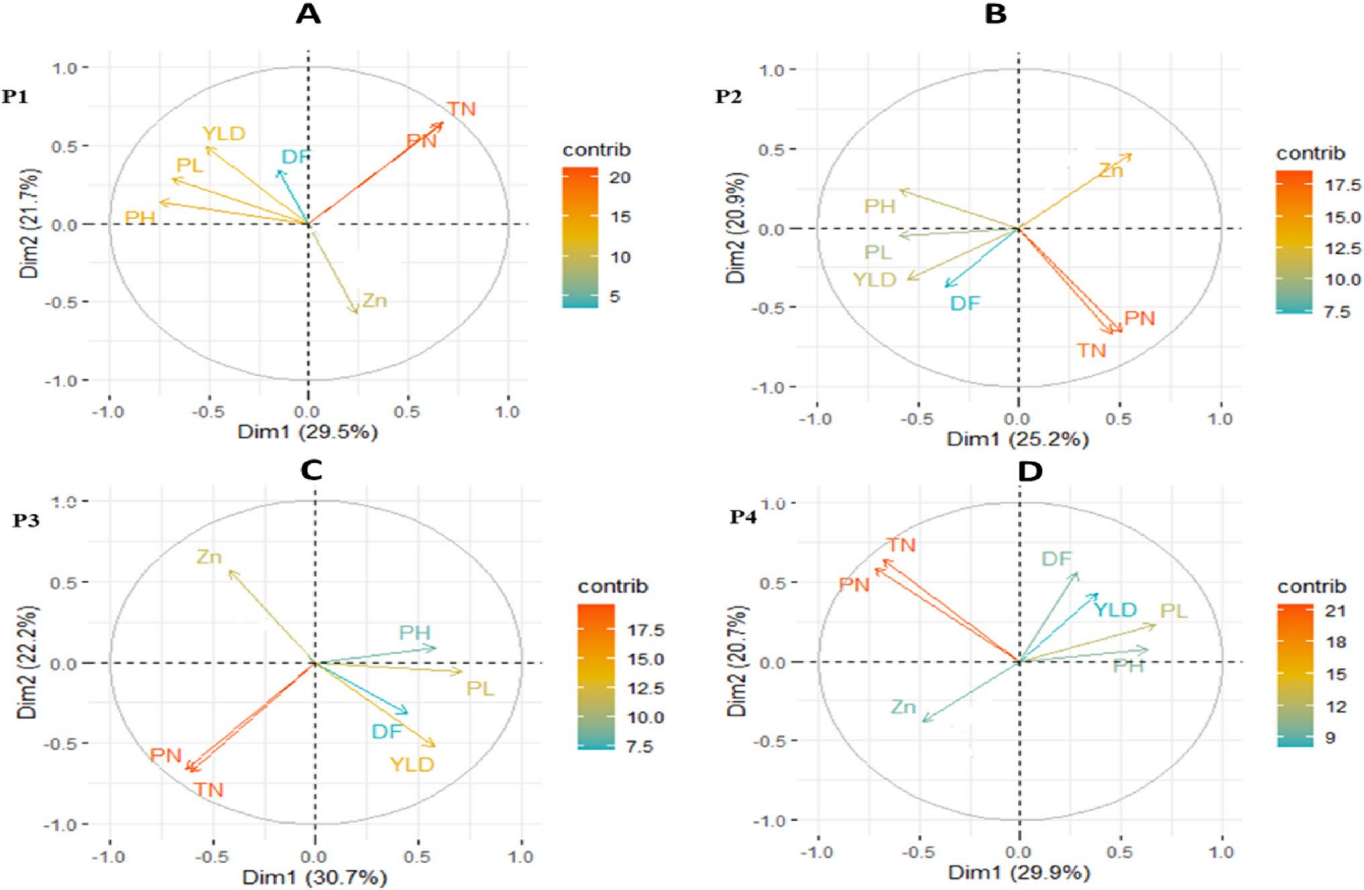
Principal component analysis of all four RIL populations using the two PCs with the highest proportion of variance. PCA for each population was estimated using R-Program. A-D indicates PCA for four populations P1–P4; The variable factor map is obtained from the PCA on agronomic traits, yield and Zn. A small angle between variables implies positive correlation, a large one suggests negative correlation, and a 90-degree angle indicates no correlation between two traits. Dim1 corresponds to PC1, and Dim2 corresponds to PC2; variables with a high contribution to the dataset variance are in red/orange whereas variable with low contribution are in blue. DF, days to flowering (days); PH, plant height (cm); TN, tiller number; PN, panicle number; PL, panicle length (cm); YLD, yield (ton/ha); Zn, zinc (ppm) (color figure online)

Results of PC analysis are presented in Table S1 and Fig. 2. The first two principal components (PC1 and PC2) had eigen value > 1, and together they explained > 46.1% of the total variation in each population. Among the traits, TN (0.67), Zn (0.55) and PL (0.71, 0.66) had the highest positive contributions to the PC1, whereas PH (− 0.75), PL (− 0.59) and PN (− 0.63, − 0.72) had negative contributions in P1–P4, respectively. Similarly, the major positive contribution for PC2 came from TN (0.65), Zn (0.47,0.56) and TN (0.64), while the negative contributions from Zn (− 0.57), TN (− 0.67, − 0.68) and Zn (− 0.38) in P1-P4, respectively.

### Linkage map construction

A thorough filtering of 7098 SNPs was conducted for each of the populations. All the monomorphic and minor allelic SNPs, and those with > 20% missing genotypes and showing segregation distortion (*P* = *0.05*) were excluded from the analyses. It resulted in a final set of 848, 211, 398, and 784 SNPs in P1-P4 respectively. The SNPs were widely distributed on all the chromosomes (Chr) with highest number of SNPs on Chr2 and Chr4 in P1 followed by Chr1 in P4. The SNPs density varied on different chromosomes and populations with an interval of 3–14 cM between the SNPs. The constructed linkage map lengths were 1632, 1535, 1701 and 1684 cMs in P1–P4, respectively (Table S2).

### ICIM QTL analysis

Inclusive composite interval mapping (ICIM) detected 56 QTLs for six agronomic traits and grain Zn content in S1 and S2 from P1–P4 (Table 2). The highest number of QTLs were mapped in P4 (24), followed by P1 (22), P2 (12) and P3 (9). Similarly, highest number of QTLs were found on chr1 (10) followed by chr7 (9), chr9 (4) and chr11 (4). A maximum of 14 QTLs were mapped for DF, followed by YLD (10), PL (6), PH (5), TN (4) and PN (4). The phenotypic variance explained (PVE) by these QTLs varied from 4.5% (*qPN*_*4.1*_) to 31.7% (*qPH*_*1.1*_) with LOD (Log_10_ of the likelihood odds ratio) values ranging from 3 to 32. The *qDF*_*7.1*_, *qDF*_*7.4*_, *qDF*_*8.1*_, *qPH*_*1.1*_, *qYLD*_*9.2,*_* qZn*_*5.1*_, *qZn*_*5.2*_, and *qZn*_*7.1*_ each explained > 10% PV. The *qDF*_*7.2*_, *qDF*_*7.4*_, *qDF*_*8.1*_, *qPH*_*1.1*_, *qPL*_*1.2*_, *qYLD*_*1.2*_, *qYLD*_*5.1*_* qZn*_*3.1*_ and *qZn*_*6.2*_ were identified in at least two populations. Most of the QTLs were identified in individual environments, whereas *qDF*_*1.1*_, *qDF*_*7.2*_, *qDF*_*8.1*_, *qPH*_*1.1*_, *qPH*_*7.1*_, *qPL*_*1.2*_, *qPL*_*9.1,*_* qZn*_*5.1*_, *qZn*_*5.2*_, *qZn*_*6.1*_ and *qZn*_*7.1*_ were identified in one or more populations and both the seasons. The *qZn*_*7.1*_ had the highest PVE (17.8%) and LOD (32) with an additive effect of > 2.3 ppm.

**Table 2:** List of QTLs identified for agronomic traits, yield and grain Zn content in RILs

QTLs	Marker Position(Mb)	Marker Interval	LOD	PVE (%)	Add	Allele	Season	Pop
*qDF*_*1.1*_	0.2–6.0	S1_194844-S1_5982772	4 & 6	5.0 & 6.6	-2 &-2.3	B	S1 & S2	P2
*qDF*_*1.2*_	38.7–39.0	S1_38652270-S1_39021641	6	9.8	1	A	S2	P3
*qDF*_*3.1*_	3.5–17.9	S3_3484027-S3_17884540	4	4.6	1.8	A	S2	P2
*qDF*_*3.2*_	31.3–32.2	S3_31348032-S3_32186401	4	6.4	1.1	A	S2	P4
*qDF*_*6.1*_	3.7–4.6	S6_3721346-S6_4577289	5	8	1.5	A	S1	P3
*qDF*_*6.2*_	7.2–7.5	S6_7191499-S6_7510502	3	5.1	1	A	S2	P4
*qDF*_*7.1*_	4.4–5.1	S7_4434376-S7_5071488	8	11.7	2.3	A	S2	P1
*qDF*_*7.2*_	5.5–6.7	S7_5526898-S7_6656052-	3 & 8	4.9 & 9.0	1.0 & 2.1	A	S2 & S1	P4 & P1
*qDF*_*7.3*_	17.0–17.2	S7_16961482-S7_17186329	7	9.8	2	A	S1	P4
*qDF*_*7.4*_	28–28.8	S7_27962775-S7_28835106	4 & 6	5.3 & 10.3	1.8 & 1 .8	A	S1	P1 & P3
*qDF*_*8.1*_	21.1–21.3	S8_21053247-S8_21337998	3 to 4	6.4 to 12.2	1.6 to 2.3	A	S1 & S2	P2 &P4
*qDF*_*9.1*_	8.8–19.7	S9_8774450-S9_19650382	5	8	-2.2	B	S1	P2
*qDF*_*11.1*_	3.8–22.9	S11_3802930-S11_22881161	4	7.5	-1.8	B	S1	P2
*qDF*_*11.2*_	23.0–23.6	S11_23023708-S11_23554090	3	5.2	-5.3	B	S1	P1
*qPH*_*1.1*_	19.3–38.3	S1_19308987-S1_38258929	9 to 19	9.2 to 31.7	-5.3 to -10.8	B	S1 & S2	P1, P2, P3 & P4
*qPH*_*3.1*_	20.2–23.4	S3_20180537-S3_23354868-	5	6	5.6	A	S2	P1
*qPH*_*5.1*_	25.5–26.2	S5_25493135-S5_26158316	4	5.3	-4.3	B	S2	P4
*qPH*_*7.1*_	5.1–6.7	S7_5526898-S7_6656052-	3 & 4	4.6 & 5.6	3.7 & 4.5	A	S2 & S1	P4
*qPH*_*8.1*_	1.5–2.9	S8_1513174-S8_2944055-	3	5	-4.1	B	S2	P4
*qTN*_*1.1*_	5.4–6.7	S1_5408523-S1_6683798	4	5.5	-0.6	B	S1	P1
*qTN*_*1.2*_	35.8–36.2	S1_35825579-S1_36205110	5	5.9	0.5	A	S1	P1
*qTN*_*2.1*_	18.9–21.5	S2_18861653-S2_21460400	4	5.3	-0.4	B	S1	P4
*qTN*_*11.1*_	18.2–18.5	S11_18229870-S11_18483298	4	6.5	-0.5	B	S1	P4
*qPN*_*2.1*_	18.9–21.5	S2_18861653-S2_21460400	5	8.1	-0.5	B	S1	P4
*qPN*_*4.1*_	22.2–22.7	S4_22179473-S4_22743183	3	4.5	-0.3	B	S2	P1
*qPN*_*5.1*_	27.7–28.7	S5_27683752-S5_28678174	3	5.4	0.3	A	S2	P1
*qPN*_*11.1*_	20.1–20.7	S11_20074439-S11_20711892	3	5.3	-1.3	B	S2	P3
*qPL*_*1.1*_	22.0–28.5	S1_22030659-S1_28478660	6	9.7	0.9	A	S2	P1
*qPL*_*1.2*_	36.7–38.2	S1_36734765-S1_38224245-	3 to 5	6.0 to 7.0	-0.5 to -0.6	B	S1 & S2	P1 & P4
*qPL*_*7.1*_	5.5–6.7	S7_5526898-S7_6656052	3	6.4	0.5	A	S1	P4
*qPL*_*7.2*_	23.5–24.3	S7_23535052-S7_24313817	3	5.1	0.5	A	S2	P4
*qPL*_*9.1*_	17.8–17.9	S9_17767194-S9_17924577	3 & 3	5.4 & 5.5	-0.4 & -0.4	B	S1 & S2	P3
*qPL*_*12.1*_	1.8–4.0	S12_1753550-S12_4046550	3	4.7	0.8	A	S2	P4
*qYLD*_*1.1*_	8.6–14.7	S1_8585951-S1_14736394	5	9.3	-0.1	B	S2	P1
*qYLD*_*1.2*_	36.7–38.2	S1_36734765-S1_38224245	3 & 4	5.8 & 5.9	-0.03 & -0.04	B	S1	P2 & P1
*qYLD*_*1.3*_	42.0–42.4	S1_41997238-S1_42408243	6	8.9	-0.09	A	S1	P1
*qYLD*_*5.1*_	0.9–19.4	S5_922530-S5_19352010	3 & 5	5.1 & 7.0	0.03 & 0.05	A	S2	P4 & P3
*qYLD*_*7.1*_	5.1–5.5	S7_5071488-S7_5526898	4	6.7	0.02	A	S1	P4
*qYLD*_*7.2*_	14.7–17.7	S7_14677901-S7_17654305	4	5.9	0.04	A	S1	P1
*qYLD*_*8.1*_	26.8–27.5	S8_26799315-S8_27517812	3	4.7	0.02	A	S1	P1
*qYLD*_*9.1*_	12.3–13.6	S9_12326525-S9_13555732	5	5.9	-0.03	B	S2	P1
*qYLD*_*9.2*_	19.2–19.7	S9_19208050-S9_19667346-	8	12.9	-0.09	B	S1	P3
*qYLD*_*10.1*_	19.1–20.4	S10_19067281-S10_20399301	3	5.4	-0.03	B	S2	P2
*qZn*_*3.1*_	3.3–18.9	S3_3313047-S3_18929885	3 & 3	4.7 & 6.7	-0.7 & -1.1	B	S1	P2 & P4
*qZn*_*3.2*_	12.1–12.7	S3_12070220-S3_12694073	3	5.2	-0.6	B	S2	P4
*qZn*_*3.3*_	13.5–13.9	S3_13456686-S3_13932387	3	6.6	-0.9	B	S2	P4
*qZn*_*4.1*_	22.2–22.8	S4_22179473-S4_22796747-	5	6.6	-0.8	B	S2	P1
*qZn*_*4.2*_	29.1–30.4	S4_29067212-S4_30393769	4	6.8	-0.7	B	S1	P4
*qZn*_*4.3*_	33.4–33.9	S4_33417482-S4_33908182	4	7.7	-0.7	B	S2	P4
*qZn*_*5.1*_	1.5–11.9	S5_1454837-S5_11897481	3 to 8	6.7 to 14.4	-0.9 to -1.5	B	S1 & S2	P2
*qZn*_*5.2*_	3.8–4.1	S5_3775755-S5_4124180	7 & 4	11.9 & 5.9	-1.2 & -0.7	B	S1 & S2	P1
*qZn*_*5.3*_	24.3–25.5	S5_24298176-S5_25493135	4	7.2	-0.7	B	S2	P4
*qZn*_*6.1*_	19.2–20.6	S6_19163386-S6_20637451	4 & 4	6.0 & 5.4	-0.8 & -0.7	B	S1 & S2	P1
*qZn*_*6.2*_	29.4–29.4	S6_29416997-S6_29448325	5 & 4	6.3 & 5.8	-0.8 & -0.7	B	S2	P2 & P1
*qZn*_*6.3*_	30.5–30.5	S6_30493399-S6_30519733	3	4.8	-0.7	B	S1	P2
*qZn*_*7.1*_	28–28.8	S7_27962775-S7_28835106	6 & 32	16.4 &17.8	-2.3 & -2.5	B	S2 & S1	P3

LOD, logarithm of the odds; PVE, phenotypic variance explained; Add-Additive effects; A, recipient parent allele, B, donor parent allele; Pop, population

**Fig. 3:**
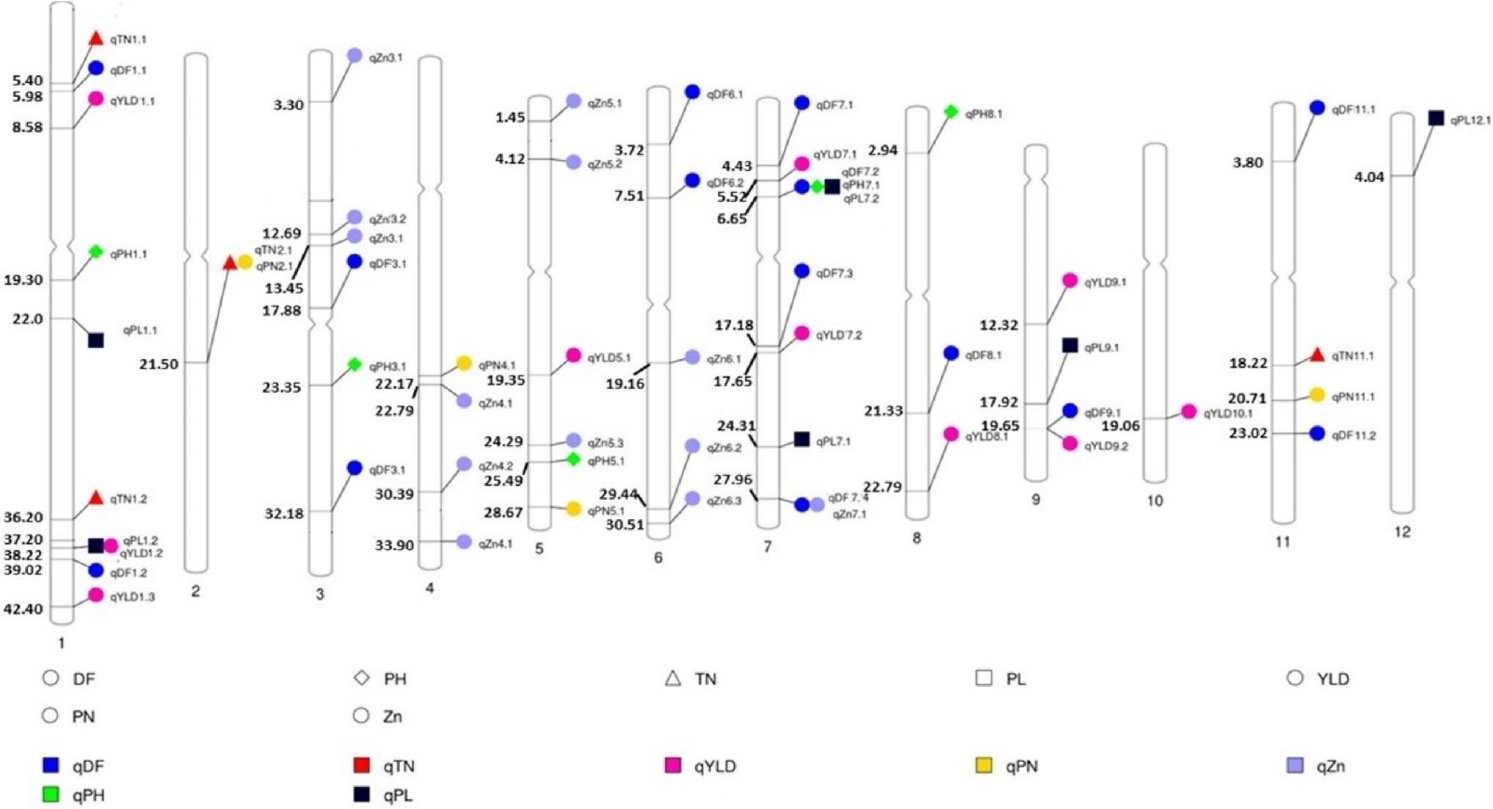
Distribution of QTLs mapped for agronomic traits, yield, and grain Zn content on all 12 chromosomes in all four populations. SNP markers were mapped to the chromosome to represent the QTLs’ physical locations (Mb). The physical position of each SNP marker was shown on the left side, with the corresponding agronomic traits, yield, and Zn displayed on the right side. QTLs of different types of traits were distinguished by different colors: blue for DF (Days to flowering), green for PH (Plant height in cm), red for TN (Tiller number), yellow for PN (Panicle number), black for PL (Panicle length in cm), pink for YLD (Yield in ton/ha), and purple for Zn (Zinc in ppm) (color figure online)

There were five chromosomal regions with two or more QTLs co-located (Fig. 3, Table 2). For example, the *qDF*_*7.2*_, *qPH*_*7.1*_ and *qPL*_*7.1*_ were co-located on chr 7 at 6.65 Mb, *qPL*_*1*.*2*_ and *qYLD*_*1*.*2*_ were co-located on chr1 at 38.22 Mb, *qTN*_*2*.*1*_ and *qPN*_*2*.*1*_ on chr2 at 21.50 Mb, *qDF*_*7.4*_ and *qZn*_*7*.*1*_ on chr7 at 27.96 Mb, whereas *qDF*_*9*.*1*_ and *qYLD*_*9*.*2*_ were co-located on chr9 at 19.65 Mb. All the QTL co-locations were observed for agronomic traits except the co-localization of *qDF*_*7.4*_ and *qZn*_*7*.*1*_.

### Epistasis

We identified 16 epistatic interactions that were either population or season specific and explained 4.5% to 11.4% of PV with a LOD > 3 (Table 3). The highest number of epistatic loci was identified for DF (5) and Zn (5) followed by YLD (3). Among five epistatic interactions of DF, four had positive additive × additive effects between loci and explained 4.9 to 11.3% PV. Single positive epistatic interactions were observed for PN on chr4/chr10 (PVE, 7%), PL chr5/chr6 (PVE, 5.8%) and YLD chr1/chr2 (PVE, 10%). Among the five interactions four were positive for grain Zn, out of which one was observed on chr1/chr2 during S1 with a high PVE of 10.5%, and also negative interaction was observed between chr1/chr2 with PVE of 11.4% during S2.

**Table 3:** Epistatic interactions identified for different traits in four mapping populations

Trait	Chr	Position (Mb)	Marker Interval	Chr	Position (Mb)	Marker Interval	LOD	PVE (%)	Add1	Add2	Add by Add	Pop	Season
DF	1	23.4–24.1	S1_23361499-S1_24115700	11	10.2–11.5	S11_11484685-S11_10190951	5	10.9	− 0.3	0.7	1.8	P1	S1
DF	1	12.7–14.0	S1_12733173-S1_13988902	1	24.4–25.2	S1_24350239-S1_25248004	5	4.9	0	0.1	1.7	P3	S1
DF	4	21.9–23.9	S4_23875094-S4_21864875	11	0.3–2.4	S11_2379158-S11_256688	5	6.8	− 1.6	− 1.1	− 3.4	P1	S2
DF	5	2.8–21.8	S5_2770834-S5_21780726	6	0.4–0.6	S6_646915-S6_400753	5	11.3	0.9	0.3	3.2	P4	S2
DF	5	6.6–9.2	S5_9214505-S5_6607675	6	2.0–26.1	S6_2010737-S6_26095829	6	6.6	− 1.8	− 1.5	2.7	P1	S2
PN	4	16.5–30.1	S4_30132071-S4_16456344	6	3.7–8.6	S6_3721346-S6_8599949	5	6.6	− 1.2	1.7	− 1.5	P4	S2
PN	4	16.5–30.1	S4_30132071-S4_16456344	10	19.7–20.3	S10_20272810-S10_19678268	6	7	− 0.8	− 1.5	1.5	P4	S2
PL	5	14.8–15.9	S5_14821944-S5_15880507	6	1.8–1.9	S6_1867779-S6_1928403	3	5.8	1.1	1.3	2	P2	S2
YLD	1	5.4–35.1	S1_35123303-S1_5354306	2	25.1–29.9	S2_25103047-S2_29934311	5	10	0	− 0.1	0.3	P2	S2
YLD	4	19.1–33.9	S4_19123643-S4_33908182	10	14.5–14.6	S10_14559932-S10_14640458	4	4.7	0	0.09	− 0.2	P2	S2
YLD	10	19.0–19.1	S10 19,020,984-S10 19,025,553	12	25.5–26.3	S12_26324348-S12_25491650	3	7.7	− 0.7	0.2	− 0.3	P2	S2
Zn	1	7.2–8.2	S1_8174333-S1_7180380	2	17.0–19.2	S2_19169013-S2_17022093	5	11.4	− 0.2	0.2	− 0.9	P4	S2
Zn	1	41.1–42.4	S1_41089199-S1_42408243	2	8.9–30.2	S2_8935514-S2_30345735	5	10.5	0.7	1.2	1.4	P1	S1
Zn	5	2.9–6.5	S5_6506422-S5_2889262	10	14.1–19.1	S10_19057844-S10_14086902	5	4.5	0.3	− 0.3	1.3	P1	S1
Zn	5	12.5–20.2	S5_20151626-S5_12458559	9	0.8–12.6	S9_12599992-S9_822892	6	8	− 1.2	− 0.9	1.7	P1	S2
Zn	10	16.6–19.0	S10_19025553-S10_16645172	11	22.1–28.0	S11_22071896-S11_27992326	3	4.6	0.6	0.4	1.2	P2	S2

Chr, chromosome; LOD, logarithm of the odds; Add-additive effects; Add by Add, additive by additive effects; Pop, population

### Association mapping

**Fig. 4:**
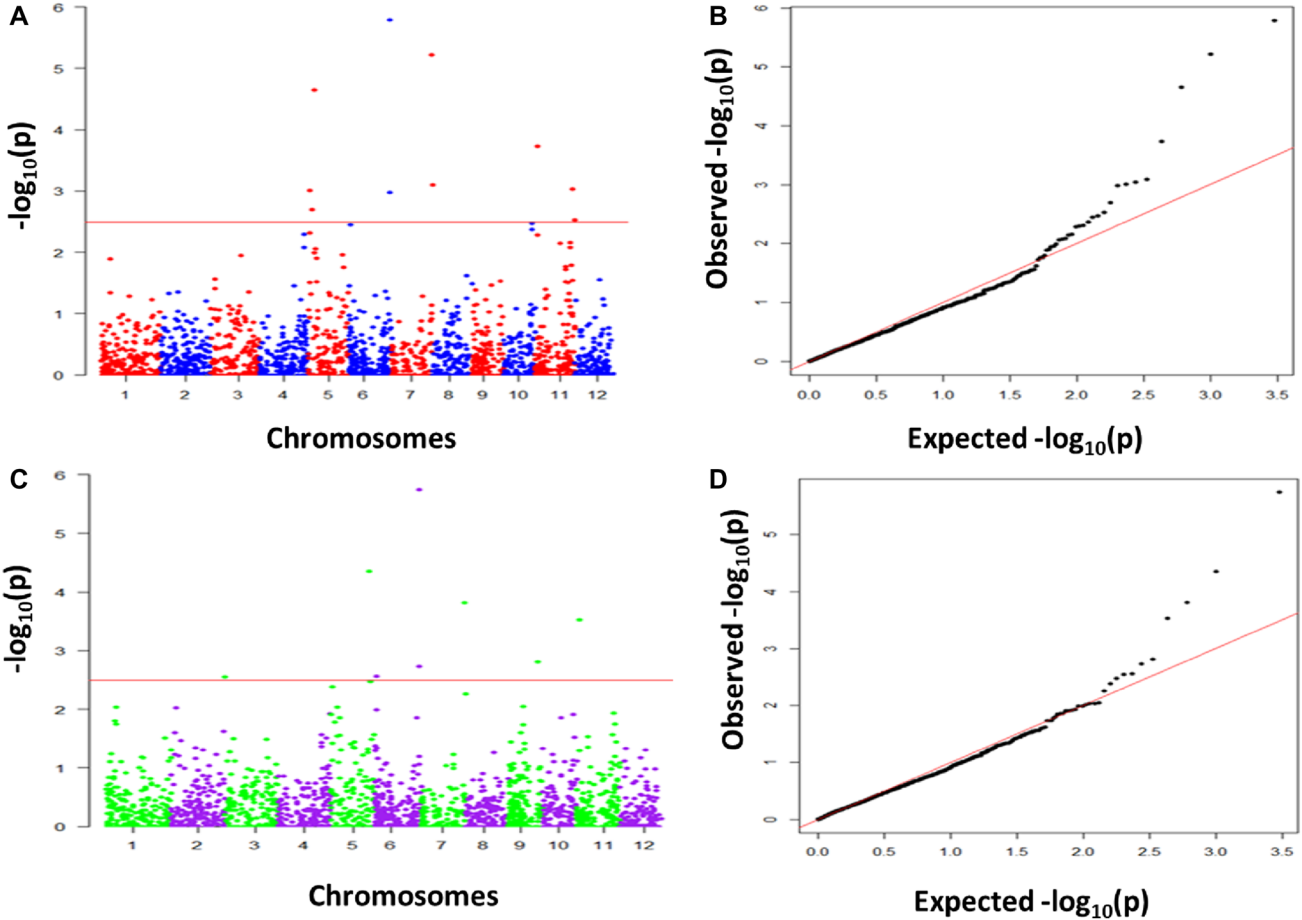
Genome-wide association analysis of grain Zn using Compressed Mixed Linear Model (CMLM) in GAPIT v3. Manhattan plots on the right display associated significant SNP markers for grain Zn detected during the 2019 wet season (**A**) and the 2020 dry season (**B**), with quantile–quantile plots for each season on the left. The X-axis represents chromosome numbers, and Y-axis represents − log 10 (*p*). The horizontal red line indicates the threshold *p*-value at significant level (*p* < 0.0001) (color figure online)

**Fig. 5:**
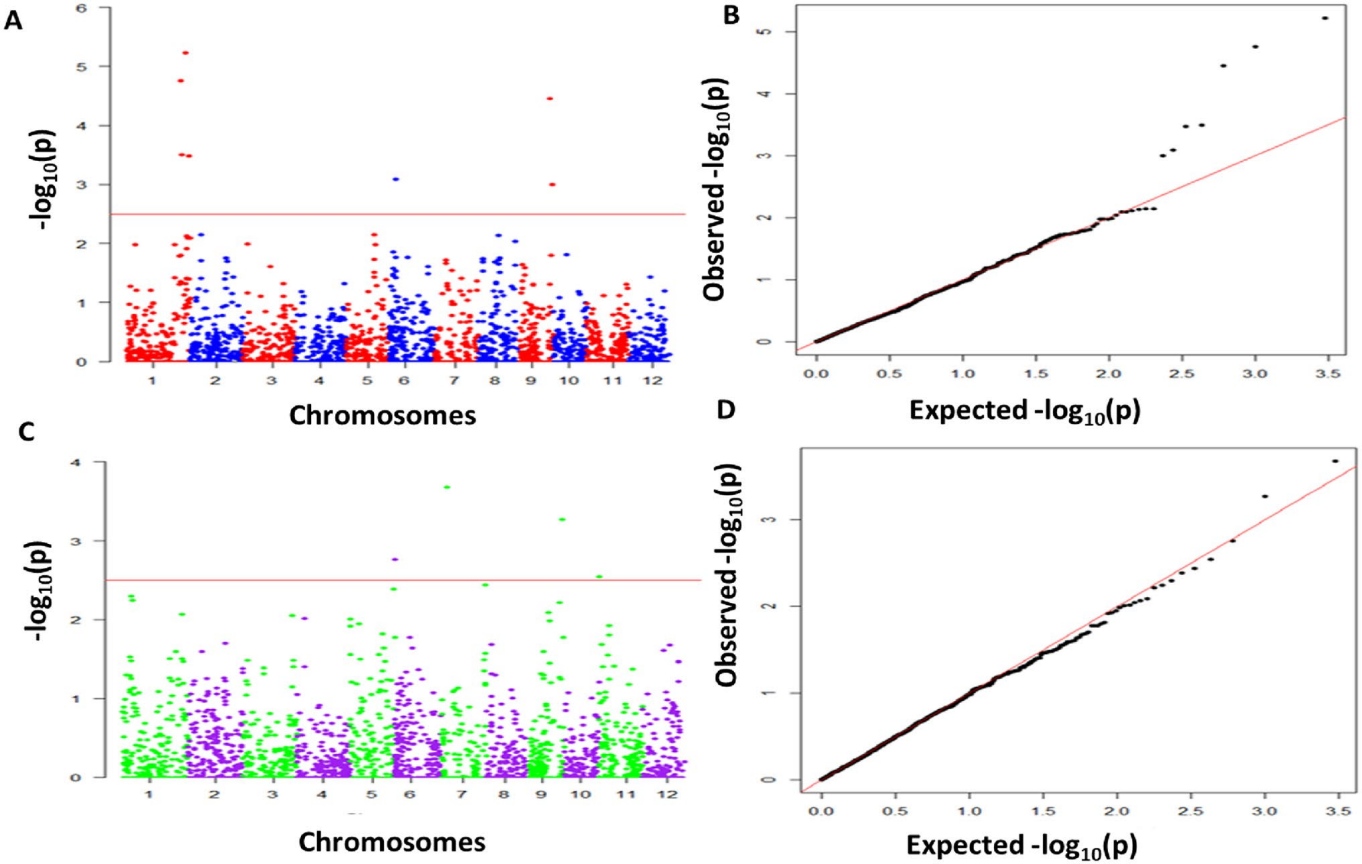
Genome-wide association analysis of YLD using Compressed Mixed Linear Model (CMLM) in GAPIT v3. Manhattan plots on the right display associated significant SNP markers for YLD detected during the 2019 wet season (**A**) and the 2020 dry season (**B**), with quantile–quantile plots for each season on the left. The X-axis represents chromosome numbers, and Y-axis represents − log 10 (*p*). The horizontal red line indicates the threshold *p*-value at significant level (*p* < 0.0001) (color figure online)

We conducted association analysis for YLD and Zn using combined data of all the four populations. In all, 2005 SNPs across the populations were used for the association analysis. A LOD threshold of 3.0 was used to declare significant marker trait associations (MTAs). Signicant MTAs were identified for grain Zn content at nine geomic regions on chromosomes 3, 5, 6, 7, 9 and 11, while MTAs for YLD were significant at seven places on chromosomes 1, 6, 7, 9 and 11 (Table 4; Figs. 4, 5). All the SNPs linked to grain Zn content had a major effect with a PVE > 18%. In contrast, all the SNPs associated with YLD had a minor effect with a PVE < 10%. The *qZn*_*5.1*_*, qZn*_*5.2*_, *qZn*_*5.3,*_* qZn*_*6.2,*_* qZn*_*7.1*_ and *qYLD*_*1.2*_ were consistently identified by both ICIM and association analyses.

**Table 4:** QTL/SNP associated with Zn content and YLD in four mapping populations

Trait	SNP	Chr	Position (Mb)	LOD	PVE (%)	Season	QTLs (ICIM)
Zn	S3_484812	3	0.48	3	17.8	S2	
	S5_1196400 -S5_4589783	5	1.19 to 4.59	3 to 5	20.1 to 24.1	S1 & S2	*qZn*_*5.1*_*, qZn*_*5.2*_
	S5_25493135	5	25.49	4	22.3	S2	*qZn*_*5.3*_
	S6_1344132	6	1.34	3	18.5	S2	
	S6_29417000 -S6_29448325	6	29.41 to 29.44	3 to 6	20.3 to 25.2	S1 & S2	*qZn*_*6.2*_
	S7_28851245- S7_29617554	7	28.85 to 29.61	3 to 4	22.8	S1 & S2	*qZn*_*7.1*_
	S9_19208050	9	19.21	3	19.4	S2	
	S11_2514115	11	2.51	3 to 4	19.3 to 22.3	S1 & S2	
	S11_27591935—S11_287395	11	27.59 to 28.75	3	21.9 to 22.1	S1	
YLD	S1_36734765—S1_37416454	1	36.73 to 37.41	4 to 5	6.9 to 7.3	S1	*qYLD*_*1.2*_
	S1_39799820—S1_41890713	1	39.79 to 41.89	3 to 5	6.1 to 6.7	S1	
	S6_4757948	6	4.75	3	6.1	S1	
	S6_400753	6	0.41	3	11.5	S2	
	S7_3853141	7	3.86	4	11.8	S2	
	S9_20587039—S9_21622536	9	20.59 to 21.62	3 to 4	6.4 to 9.6	S1 & S2	
	S11_933288	11	0.93	3	10.0	S2	

Chr, chromosome; LOD, logarithm of the odds; PVE, phenotypic variance explained

### In-silico candidate gene and co-expression analysis

We conducted in-silico candidate gene analysis of *qZn*_*6.2*_. There were 11 genes within 50 kb vicinity of consistent peak SNP (S6_29448325) such as CLKC kinase, macrophage migration inhibitory factor, ANTH/ENTH domain containing protein, CCT motif containing protein, 4-amino-4-deoxy chorismate synthase, proteasome/cyclosome repeat containing protein, *OsSub52-*putative subtilisin homologue, dDefensin-like DEFL family, expressed protein, DNA biniding protein, transposon protein and cadmium/zinc-transporting ATPase 4.

**Fig. 6:**
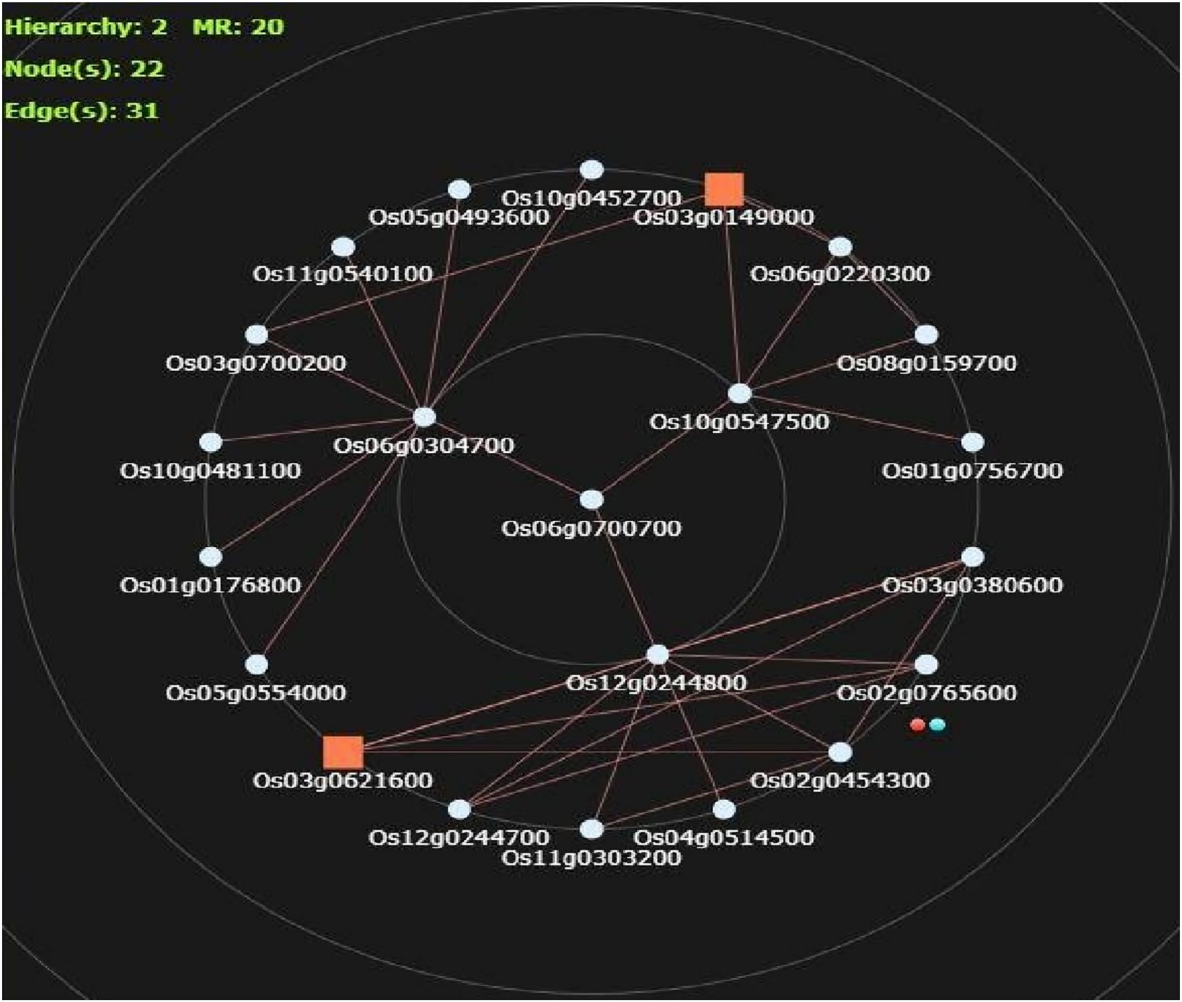
HyperTree depicting co-network analysis of predicted candidate gene *LOC*_*Os06g0700700.* The figure shows various coexpressed genes associated with *LOC*_*Os06g0700700* in a HyperTree format using RiceFREND. Transcription factor encoding genes are represented as orange square boxes, and the candidate gene *LOC*_*Os06g0700700* is highlighted at the center of the circular HyperTree

Twenty genes were co-expressed with *OsHMA2* (Fig. 6). Among them *OsKAT1, OsAmy1A, Lsi3/SIET2* and *OsMATE2* are well characterized, while the remaining are conserved hypothetical proteins, unknown proteins, eggshell protein family, aquaporin protein, transcriptional factor B3 domain containing protein, pentatricopeptide repeat domain containing protein and indole-3-acetate beta-glucosyltransferase. The *OsKAT1* codes for shaker potassium channel protein responsible for salinity stress tolerance and ion homeostasis*, OsAmy1A* codes for α-amylase glycoprotein that is responsible for the degradation of starch granule, *Lsi3/SIET2* codes for Silicon efflux transporter (SIET/Lsi2-like) homolog which assists in efflux intravascular transport of silicon and *OsMATE2* codes for multidrug and toxic compound extrusion (MATE) protein family transporter responsible for the arsenic stress response, regulation of plant growth and development, and disease resistance.

### QTL classes for Zn and YLD

The pyramiding effects of YLD and Zn QTLs is provided in the Table 5. We observed an increase in grain Zn content and yield with increase in number of grain Zn and YLD QTLs respectively. There were 18 Zn QTL classes in four populations. Single or four Zn QTL classes had 3.32 ppm more Zn content than *qZn*_*5.2*_ class in P1. Two (16.84 ppm) and three QTLs (17.07 ppm) classes had higher Zn content in P2, while there was only one Zn QTL (12.05 ppm) in P3. The lines with *qZn*_*5.3*_ had a mean grain Zn content of 23.67 ppm and performed better than lines with two Zn QTLs, but three QTL classes had mean grain Zn content of > 26.63 ppm. The *qZn*_*5.1*_, *qZn*_*5.2*_ and *qZn*_*5.3*_ are the major contributors to increased grain Zn content either individually or in combination with other Zn QTLs in different populations. The three QTL class (*qZn*_*5.1*_ + *qZn*_*6.2*_ + *qZn*_*6.3*_) had highest grain Zn content of 28.02 ppm and had a highest Zn increment of 17 ppm in P3 in comparison to other QTL classes in different populations. Similarly, there were 17 YLD QTL classes in four populations (Table 5). The *qYLD*_*1.2*_ (4.82 t/ha, 5.33 t/ha), *qYLD*_*1.2*_ + *qYLD*_*1.3*_ (5.27 t/ha), *qYLD*_*1.2*_ + *qYLD*_*10.1*_ (5.61 t/ha) and *qYLD*_*1.1*_ + *qYLD*_*1.2*_ + *qYLD*_*1.3*_ (5.57 t/ha) classes had highest grain yield in their respective groups either in P1 or P2, but *qYLD*_*1.2*_ is the major contributor to yield increase in these two populations. While, *qYLD*_*5.1*_ is the major contributor and had highest effect on yield increase in P3 and P4.

**Table 5:** Effects of QTL classes for grain Zn content and YLD

QTL class	Zn (ppm)/YLD (t/ha)	Difference with no QTL	Pop
None	11.06^d^	0	P1
*qZn*_*4.1*_	18.78^c^	7.72	
*qZn*_*5.2*_	22.34^ab^	11.28	
*qZn*_*6.1*_	21.27^ab^	10.21	
*qZn*_*4.1*_ + *qZn*_*5.2*_ + *qZn*_*6.1*_ + *qZn*_*6.2*_	25.67^a^	14.61	
None	10.95^d^	0	P2
*qZn*_*5.1*_	23.51^ab^	12.56	
*qZn*_*6.3*_	20.49^bc^	9.54	
*qZn*_*6.3*_ + *qZn*_*5.1*_	27.79^a^	16.84	
*qZn*_*5.1*_ + *qZn*_*6.2*_ + *qZn*_*6.3*_	28.02^a^	17.07	
None	11.23^b^	0	P3
*qZn*_*7.1*_	22.48^a^	12.05	
None	11.98d	0	P4
*qZn*_*3.1*_	17.56^cd^	5.58	
*qZn*_*3.3*_	16.67^cd^	4.69	
*qZn*_*4.1*_	18.02^c^	6.04	
*qZn*_*4.2*_	17.23^cd^	5.25	
*qZn*_*5.3*_	23.67^c^	11.69	
*qZn*_*3.3*_ + *qZn*_*3.1*_	18.65^c^	6.67	
*qZn*_*4.3*_ + *qZn*_*5.3*_	23.01^ab^	11.03	
*qZn*_*3.2*_ + *qZn*_*3.3*_ + *qZn*_*5.3*_	26.63^a^	14.65	
*qZn*_*4.1*_ + *qZn*_*4.2*_ + *qZn*_*5.3*_	27.13^a^	15.15	
None	4.19^e^	0	P1
*qYLD*_*1.2*_	4.82^d^	0.63	
*qYLD*_*7.1*_	4.56^ed^	0.37	
*qYLD*_*8.1*_	4.33^e^	0.14	
*qYLD*_*1.1*_ + *qYLD*_*1.2*_	4.91^cd^	0.72	
*qYLD*_*1.2*_ + *qYLD*_*1.3*_	5.27^ab^	1.08	
*qYLD*_*1.1*_ + *qYLD*_*7.2*_	4.82^d^	0.63	
*qYLD*_*1.1*_ + *qYLD*_*1.2*_ + *qYLD*_*7.2*_	5.22^ab^	1.03	
*qYLD*_*1.1*_ + *qYLD*_*1.2*_ + *qYLD*_*1.3*_	5.57^a^	1.38	
*qYLD*_*1.1*_ + *qYLD*_*1.2*_ + *qYLD*_*1.3*_ + *qYLD*_*8.1*_	5.49^a^	1.30	
*None*	4.73^b^	0	P2
*qYLD*_*1.2*_	5.33^ab^	0.60	
*qYLD*_*10.1*_	4.91^b^	0.18	
*qYLD*_*1.2*_ + *qYLD*_*10.1*_	5.61^a^	0.88	
*None*	4.81^b^	0	P3
*qYLD*_*5.1*_	5.53^a^	0.72	
*qYLD*_*9.2*_	5.13^ab^	0.32	
*None*	3.92^b^	0	P4
*qYLD*_*5.1*_	4.62^a^	0.70	
*qYLD*_*7.1*_	4.57^a^	0.65	
*qYLD*_*5.1*_ + *qYLD*_*7.1*_	4.69^a^	0.77	

Pop, population; Different letters indicate significant differences at *P* < 0.05

### Genomic prediction for grain Zn and yield

**Fig. 7:**
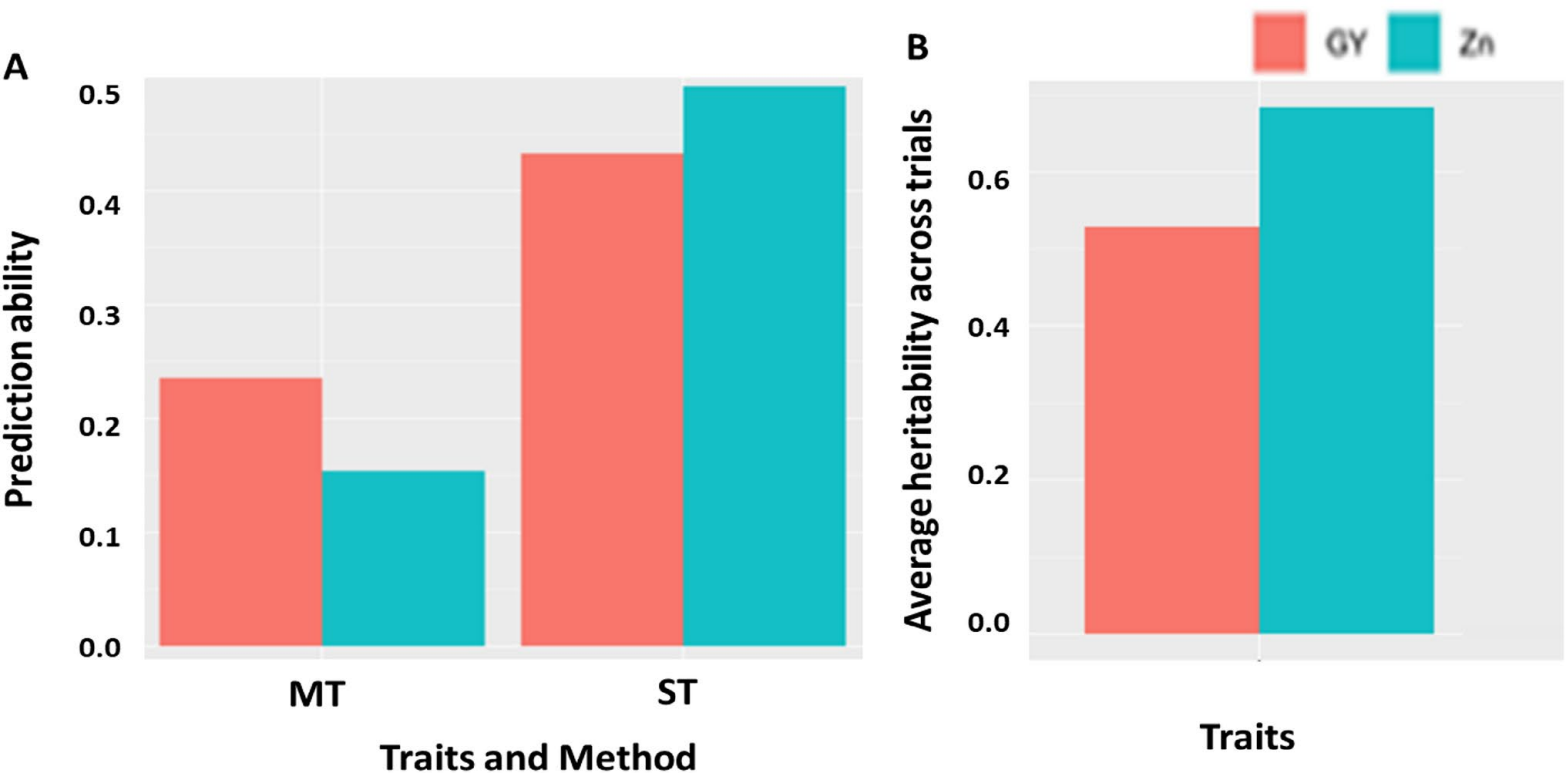
Graphical depiction of prediction accuracy and heritability. Genomic prediction was estimated through single-trait (YLD/Zn individually) and multi-trait (YLD and Zn combined) strategies for all populations. MT, multi-trait; ST, single trait, Linear mixed models were implemented via ASReml-R (Analysis Software for Residual Maximum Likelihood following a CV-alpha) using a cross-validation system with four replicates and 5-folds. Deregressed Best Linear Unbiased Predictors (dBLUPs) were utilized as the response variable for each trait

The genomic prediction results of YLD and grain Zn content are depicted in Figs. 7a, b. The average broad-sense heritability for YLD recorded with 0.54, whereas Zn content was 0.69, across the populations and seasons. The multi-trait model prediction accuracy results for YLD and grain Zn were 0.24 and 0.16, respectively. Interestingly, the single-trait prediction model resulted in an accuracy of 0.44 and 0.49 for YLD and Zn, respectively.

### Identification of lines with high Zn and YLD

Five best lines were selected from each population based on grain Zn content (≥ 24 ppm) and YLD (≥ 5.0 t/ha), across the seasons (Table 6). All the lines possessed different grain Zn QTL combinations, which were mapped in this study; interestingly in P3 all the genotypes possessed single QTL *qZn*_*7.1*_ and in P1 two genotypes had *qZn*_*5.2*_. The selected genotype's grain Zn content and YLD ranged from 22.57 ppm to 29.17 ppm and 4.23 t/ha to 6.38 t/ha, respectively, across the seasons and populations. In P1 grain Zn and YLD ranged from 22.57 to 28.22 ppm and 4.23 to 6.36 t/ha, 23.18 to 28.10 ppm and 4.31 to 6.02 t/ha in P2, 24.22 to 28.5 ppm and 4.31 to 6.38 t/ha in P3, and 23.80 to 29.17 ppm and 4.47 to 6.32 t/ha in P4 were recorded (Table 6). Whereas the grain Zn content and YLD of the recipient parents (RP) ranged from 10.2 to 11.1 ppm and 3.2 to 5.4 t/ha respectively. While donor parents (DP) had 27.6 to 34.4 ppm of grain Zn content and 1.5 to 3.1 t/ha YLD (Table 1). All the selected lines had > 15 ppm of advantage of grain Zn and YLD comparable to RPs. Whereas, the genotypes IR 133350-B-260-1-B, IR 133350-B-453-1-B and IR 133362-B-586-2-B contains high Zn content with > 27 ppm.

**Table 6:** Best genotypes with high Zinc and yield among the populations

Pop	Designation	Zn (ppm)		YLD (t/ha)		QTLs for Zn
		S1	S2	S1	S2	
P1	IR133350-B-123-1-B	28.12	26.32	5.31	5.01	*qZn*_*4.1*_*, qZn*_*5.2*_*, qZn*_*6.1*_*, qZn*_*6.2*_
	IR 133350-B-260-1-B	27.42	27.6	5.91	6.15	*qZn*_*4.1*_*, qZn*_*5.2*_*, qZn*_*6.1*_*, qZn*_*6.2*_
	IR133350-B-155-2-B	25.05	24.87	5.64	4.51	*qZn*_*5.2*_
	IR 133350-B-453-1-B	27.73	28.22	6.36	4.23	*qZn*_*4.1*_*, qZn*_*5.2,*_* qZn*_*6.1*_*, qZn*_*6.2*_
	IR133350-B-63-2	25.25	22.57	5.90	4.87	*qZn*_*5.2*_
P2	IR 133353-B-288-2-B	24.35	25.95	5.03	6.02	*qZn*_*5.1*_*, qZn*_*6.2*_
	IR 133353-B-349-1-B	27.02	26.1	5.41	5.93	*qZn*_*5.1*_*, qZn*_*6.2*_*, qZn*_*6.3*_
	IR 133353-B-316-2-B	23.93	23.18	5.90	4.31	*qZn*_*5.1*_*, qZn*_*6.2*_
	IR 133353-B-283-1-B	28.10	25.55	5.21	4.6	*qZn*_*5.1*_*, qZn*_*6.2*_*, qZn*_*6.3*_
	IR 133353-B-360-2-B	26.45	23.35	5.92	5.67	*qZn*_*5.1*_*, qZn*_*6.2*_*, qZn*_*6.3*_
P3	IR133359-B-410-2-B	24.22	25.2	5.62	5.11	*qZn*_*7.1*_
	IR133359-B-602-1-B	24.47	25.9	5.91	5.61	*qZn*_*7.1*_
	IR133359-B-275-1-B	28.5	26.73	6.29	4.24	*qZn*_*7.1*_
	IR133359-B-282-2-B	25.55	23.72	6.38	4.31	*qZn*_*7.1*_
	IR133359-B-337-2-B	27.00	28.45	5.25	4.55	*qZn*_*7.1*_
P4	IR 133362-B-586-2-B	29.17	27.77	5.64	5.07	*qZn*_*4.3*_*, qZn*_*4.2*_*, qZn*_*5.3*_
	IR 133362-B-483-1-B	28.47	24.45	4.47	6.32	*qZn*_*4.3*_*, qZn*_*4.2*_*, qZn*_*5.3*_
	IR 133362-B-379-1-B	25.82	25.63	4.84	6.23	*qZn*_*3.1*_*, qZn*_*3.3*_*, qZn*_*5.3*_
	IR 133362-B-630-2-B	23.97	26.28	6.23	5.07	*qZn*_*4.3*_*, qZn*_*5.3*_
	IR133362-B-298-1-B	23.80	24.20	5.04	5.53	*qZn*_*4.3*_*, qZn*_*5.3*_

_Pop, population_

## Discussion

Zinc malnutrition remains a major health concern in the developing world due to the monotonous consumption of less nutritious cereals and lack of dietary diversity (Arfi et al. 2022; Wessells and Brown 2012). Biofortified crop varieties are cheaper, easily accessible, and sustainable sources of daily nutrition (Bouis and Saltzman 2017; Nuthalapati et al. 2022; Pradhan et al. 2020). Even though several high Zn rice varieties have been released in different countries of Asia, there is a need to enhance the grain Zn content, to broaden the scope of Zn breeding to other Asian countries and fast-track Zn mainstreaming in rice (Bouis and Saltzman 2017; Calayugan et al. 2021). Therefore, understanding the genetic basis of grain Zn content and associated agronomic and yield traits could help breed Zn enriched rice varieties. In the present study we dissected genetic factors responsible for high grain Zn content, agronomic and yield traits using four *aus* derived bi-parental populations.

Several studies have reported the importance of *aus* accessions in breeding for stress tolerance and nutritional improvement in rice (Inabangan-Asilo et al. 2019; Palanog et al. 2023; Rakotondramanana et al. 2022). The *aus* accessions are rich source of grain Zn content but they have been less explored in molecular characterization to identify QTLs/genes for grain Zn conent in rice (Rakotondramanana et al. 2022; Swamy et al. 2018). The IRRI rice gene bank holds > 3000 *aus* accessions. Our efforts to systematically characterize them for grain Zn content and yield potential resulted in identification of several promising high Zn donor accessions such as Kaliboro (IRGC77201-1), Jamir (IRGC117765), UCP122 (IRGC8794-1), Lalsaita (IRGC43915-1) etc. (Palanog et al. 2023; Rakotondramanana et al. 2022; Swamy et al. 2018). We developed four RILs populations using high Zn donor accessions UCP122 and Lalsaita, and characterized them for agronomic, yield and grain Zn content traits for two seasons at IRRI. Both the donor accessions had two to three folds more grain Zn content than the recipient parents. All the populations showed wider variations and transgressive segregants with highly significant genotypic effects and modest to high heritability for almost all the traits across the seasons and populations (Table 1). These results are in consonance with earlier reports on traits distributions, genotypic effects and heritability (Calayugan et al. 2020; Descalsota et al. 2019a; Singhal et al. 2021; Suman et al. 2021). The parental lines of the RILs showed moderate marker polymorphism with a rate of > 55% between the parents and a very few segregation distortortion (Rahman et al. 2017; Sasaki 2005; Wen et al. 2020).

We mapped a total of 56 QTLs for seven traits through ICIM in four populations, as expected the highest number of QTLs were identified for DF (14), Zn (13) and YLD (10). However, majority of the minor effect QTLs were identified in a specific season or population. *qDF*_*7.1*_, *qDF*_*7.4*_, *qDF*_*8.1*_, *qPH*_*1.1*_, *qYLD*_*9.2,*_* qZn*_*5.1*_, *qZn*_*5.2*_, and *qZn*_*7.1*_ each explained a PV > 10%. The *qPH*_*1.1*_ is the only locus identified in both the seasons and all the populations and co-located at “*sd1*” on chromosome 1 (Bhuvaneswari et al. 2020; Yu et al. 2020). Interestingly, at 59% of the loci trait enhancing QTL alleles were contributed by the donor parents. It implies that *aus* accessions are a rich source of novel alleles for grain Zn, yield and yield related traits. *qZn*_*5.1*_*, qZn*_*5.2*_*, qZn*_*5.3*_*, qZn*_*6.2*_*, qZn*_*7.1*_ and *qYLD*_*1.2*_ were identified by both ICIM and association analysis (Symonds et al. 2005). *qZn*_*7.1*_ had the highest PV (17.8%) and additive effect (2.5 ppm) followed by *Zn*_*5.1*_ having a PV of 14.4% and additive effect of 1.5 ppm. *qZn*_*5.1*_, *qZn*_*5*.2,_ *qZn*_*5.3*_ and *qZn*_*7*.1_ were consistently reported from *aus* accessions derived populations and found to contribute upto 3 ppm grain Zn content (Calayugan et al. 2020; Descalsota et al. 2019a, b; Zaw et al. 2019). Several previous studies reported that Zn donors from *aus* sub-species of rice can be potential source of QTLs, genes and haplotypes for improving the grain Zn content of rice (Babu et al. 2020; Calayugan et al. 2020; Descalsota et al. 2019a; Joshi et al. 2023; Rakotondramanana et al. 2022).

Epistasis plays an important role in phenotypic expression of traits. QTL epistatic effects can be as large as individual main effect QTLs, and can also occur between minor effect QTLs (Norton et al. 2010). Therefore, dissecting epistatic effects of major QTLs is essential prior to their deployment in the breeding programs (Calayugan et al. 2020). We identified 16 epistatic interactions for DF, PN, PL, YLD and Zn content, their PVE ranged from 4.5 to 11.4%. The highest number of epistatic interactions were observed for DF and Zn (5) followed by YLD (3) (Table 3). All the observed epistatic interactions except one involving *qZn*_*5.1*_ were independent, similar results were reported in earlier studies (Descalsota et al. 2019a; Singhal et al. 2021; Uttam et al. 2023). Epistatic interactions between QTLs for Zn and PH affected their phenotypes, and also genetic background found to influence the epistatic effects that led to variable phenotypic expression (Islam et al. 2020; Jiang et al. 2008; Lekklar et al. 2019; Xu et al. 2017). Thus, epistatic effect of major QTLs and their intreactions with the genetic backgrounds should be verified before using them in the breeding programs (Calayugan et al. 2020; Islam et al. 2020; Lu et al. 2009).

Dissection and validation of major effect QTLs through fine mapping, insilico candidate gene analyses and use of gene specific markers can further improve the accuracy and precision of genomics assisted breeding. It also helps in shortlisting candidate genes for further molecular characterization (Zhu and Zhao 2007). With the recent advancements in rice genomics, post QTL analyses are routinely carried out using insilico tools to understand the genetic bases of QTLs and to deduce the candidate genes. Several studies have thoroughly characterized major effect Zn and Fe QTLs to list candidate genes and to imply their role in metal homeostasis and biofortification (Raza et al. 2019; Sasaki et al. 2015; Satoh-Nagasawa et al. 2012; Swamy et al. 2016, 2018). In this study we specifically tragetted *qZn*_*6.2*_ a relatively a novel QTL for insilico characterization. In all there were 11 different genes within 50 kb region of *qZn*_*6.2*_ but *LOC_Os06g48720* is the most probable candidate gene as it encodes for cadmium/zinc-transporting ATPase4 proteins that helps in Zn transport. *OsHMA* family genes such as *OsHMA1, OsHMA2* and *OsHMA3* are known to regulate Zn uptake and translocation in rice (Huang et al. 2022; Mu et al. 2021; Sasaki et al. 2014). The*OsHMA2* that expresses in the plasma membrane of root pericycle-cells and phloem contributes to preferential distribution of Zn in the developing tissues, and also plays a key role in the inter-vascular transfer of Zn and Cd (Sasaki et al. 2014; Satoh-Nagasawa et al. 2012; Takahashi et al. 2012b; Yamaji et al. 2013). Insilico expression analyses results showed that *OsHMA2* expressed in root, phloem cells, inflorescence, anther, pistil, lemma, palea, ovary, and embryo indicating its role in the remobilization of Zn at the time of flowering and seed maturation stages (Figure S1). Several genes/gene families such as *OsNAS, OsIRT, OsZIP OsYSL, OsNRAMP, OsNAAT, OsVIT, OsIDEF, OsMTP. OsIRT, OsOPT, OsHMA* etc. play pivotal role in Zn homeostasis from Zn absorption to transportation from roots to grain through translocation, remobilization and loading (Mu et al. 2021; Sasaki et al. 2014, 2015; Satoh-Nagasawa et al. 2012; Takahashi et al. 2012a; 2012b; Tan et al. 2019). The co-location of metal homeostasis genes with Zn QTLs provide positive evidence for the accuracy of mapping and usefulness of these QTLs in genomics assisted breeding for improved grain Zn content in rice (Mohiuddin et al. 2020).

Identification of superior QTL classes, capturing and fixing the positive QTL × QTL, and QTL × background interactions in early generations can improve the efficiency of marker assisted breeding programs (Kumar et al. 2018; Vikram et al. 2015). In this study we identified superior QTL classes for both grain Zn and YLD. In general there was an increase in YLD and Zn with increase in number of respective QTLs in the RILs. The *qZn*_*5.1*_, *qZn*_*5.2*_ and *qZn*_*5.3*_ are the major contributors to increased grain Zn content either individually or in combination with other Zn QTLs in different populations. Similary, *qYLD*_*1.2*_ is the major contributor to yield increase in P1 and P2, while, *qYLD*_*5.1*_ is the major contributor and had highest effect on yield increase in P3 and P4. However, there were specific QTL combinations/classes that were superior over others in different populations (Table 5). So, choosing better combination of QTLs for marker assisted QTL pyramiding is necessary to achive better results.

Genomic selection that captures genome wide information for selection of best plants has become intergral part of the breeding programs to achieve better genetic gains within short time and with less cost (Zaghum et al. 2022). The accurate genomic prediction for single trait is easier than multi-trait analysis because of the complex nature and negative correlation between traits. The single trait model exhibited more than two folds and three folds higher accuracy for YLD and grain Zn over the multi-trait model to select the best plants. Thus, it is clear that the multi trait analysis reduced the prediction ability of grain YLD and Zn (Arojju et al. 2020; Rakotondramanana et al. 2022). Even though different multi-trait analyses are effective but not always superior over single-trait models (Muvunyi et al. 2022). Therefore, considering the correlation between traits and the desired genetic gain to predict the grain Zn and YLD, it is better to follow single-trait based predictions. Then, based on the estimated breeding values for Zn and YLD, a selection index can be built for recycling the lines as parents in the crossing program (Pešek and Baker 1969).

The simultaneous improvement of two or more complex traits in a breeding program depends on the genetic correlation between the traits. Positively related traits can be simultaneously improved through indirect selection, while negatively correlated traits need specific breeding strategies (Dhurai et al. 2016). YLD is usually negatively correlated with grain Zn, (Ata-Ul-Karim et al. 2022; Babu et al. 2020; Calayugan et al. 2020) but there are also contrasting reports on the association of grain Zn with YLD and agro-morphological traits (Calayugan et al. 2020; Gangashetty et al. 2013; Vikram et al. 2015; Wissuwa et al. 2008). In our study, grain Zn and YLD was also negatively correlated, and their contributions to total variations was in opposite directions (Figs. 1, 2). Agronomic traits such as DF, PH, PL positively contributed to YLD indicating their importance in selection for better yield (Calayugan et al. 2020). Thus, selecting these traits with high Zn and acceptable yield potential is essential to select transgressive segregants for developing commercially viable Zn biofortified rice varieties. We identified several RILs having ≥ 24 ppm evaluated over two seasons with yield similar to recipient parents in four RILs populations. The top five genotypes selected based on YLD and grain Zn from each population showed promising levels of grain Zn (≥ 24 ppm) and acceptable yield (> 5.0 t/ha) (Table 6). These lines can be tested for varietal release or used as donors in the Zn breeding programs (Calayugan et al. 2020; Rana et al. 2019; Singh et al. 2018).

## Conclusion

Breeding Zn biofortified crops help tackling Zn malnutrition in the developing world. Genetic analyses of rice panels and populations to identify high Zn donors, QTLs and genes help fast tracking the development of Zn biofortfied rice varieties. In this study we characterized four biparental populations for agronomic, YLD and Zn traits. All the traits showed wider variations, significant genotypic effects and moderate to high heritabilities. ICIM identified 56 QTLs for agronomic, YLD, and Zn traits, and 16 MTAs were detected for YLD and Zn by association analyses. The PV explained by these QTLs varied from 4.5% (*qPN*_*4.1*_) to 31.7% (*qPH*_*1.1*_). *qDF*_*1.1*_, *qDF*_*7.2*_, *qDF*_*8.1*_, *qPH*_*1.1*_, *qPH*_*7.1*_, *qPL*_*1.2*_, *qPL*_*9.1,*_* qZn*_*5.1*_, *qZn*_*5.2*_, *qZn*_*6.1*_ and *qZn*_*7.1*_ were identified in one or more populations and in both the seasons. *qZn*_*7.1*_ had the highest PVE (17.8%), LOD (32) and additive effect of (2.5) ppm. *qZn*_*5.1*_*, qZn*_*5.2*_, *qZn*_*5.3,*_* qZn*_*6.2,*_* qZn*_*7.1*_ and *qYLD*_*1.2*_ were consistently identified by both ICIM and association analyses. There were five chromosomal regions with two or more QTLs co-located. We observed increased YLD and Zn with pyramiding of respective QTLs. In all 16 epistatic loci were identified for DF, PN, PL, YLD and Zn across the populations. The multi-trait model prediction accuracy results for YLD and grain Zn were 0.24 and 0.16, respectively. Promising genotypes exhibited high grain Zn ≥ 24 ppm with optimum levels yield ≥ 5.0 t/ha. Superior QTL classes were identified with high grain Zn and YLD, such combinations of QTLs are useful for varietal improvement. Over all, our results are useful for efficient Zn mainstream breeding of rice.

## Supplementary Information

Below is the link to the electronic supplementary material.

Supplementary file1 (DOCX 249 KB)

## Data Availability

Data is included in the article/supplementary material.

## Abbreviations

Amy1A: Amylase
ANOVA: Analysis of variance
BLUP: Best linear unbiased prediction
BLUE: Best linear unbiased estimates
CTAB: Cetyltrimethylammonium bromide
CV: Co-efficient of variation
CMLM: Compressed mixed-linear model
DF: Days to flowering
DMAS: Deoxy mugineic acid synthase
GS: Genomic selection
GWAS: Genome wide association study
HMA: Heavy metal associated proteins
HSD: Turkey’s honest significance test
ICIM: Inclusive composite interval mapping (ICIM)
IDEF: Iron deficiency-responsive cis-acting element binding factors
IRRI: International Rice Research Institute
KAT1: Rice shaker potassium channel
LOD: Logarithm of odds
MATE: Plant multi-drug and toxic compound extrusion
MQTL: Meta QTL
MTA: Marker Trait Association
MTP: Metal transport protein
NAAT: Nicotianamine amino transferase
NAS: Nicotianamine synthase
NCT: National co-operative test
NRAMP: Natural resistance-associated-macrophage protein
NT: Number of tillers
NP: Number of panicles
OPT: Oligo peptide transport proteins
PCA: Principal component analysis
PH: Plant height (cm)
PL: Panicle length (cm)
PreNCT: Pre national co-operative test
PV: Phenotypic variance
QTL: Quantitative trait loci
RGA: Rapid generation advance
RIL: Recombinant inbred lines
SAMS: S-adenosyl methionine synthase
SNP: Single nucleotide polymorphism
S1: Season 1
S2: Season 2
P1: Population 1
P2: Population 2
P3: Population 3
P4: Population 4
TOM: Transporter of mugineic acid
VIT: Vacuole iron transporter
XRF: X-ray fluorescence
YSL: Yellow stripe1-like
ZIP: ZRT, IRT like protein
YLD: Grain yield (kg/ha)
Zn: Zinc/grain zinc (ppm)

## References

[CR1] Aggett PJ (2020) Chapter 22: Iron. In: Marriott BP, Birt DF, Stallings VA, Yates AABT (eds) Present knowledge in nutrition, 11th edn. Academic Press, London, pp 375–392

[CR2] Arfi N, Khatoon K, Alim F (2022) Zinc malnutrition in children and its consequences on health. In: Tabrez S, Khan AM (eds) Microbial biofertilizers and micronutrient availability: the role of zinc in agriculture and human health. Springer, Cham, pp 35–67. 10.1007/978-3-030-76609-2_2

[CR3] Arojju SK, Cao M, Trolove M, Barrett BA, Inch C, Eady C, Stewart A, Faville MJ (2020) Multi-trait genomic prediction improves predictive ability for dry matter yield and water-soluble carbohydrates in perennial ryegrass. Front Plant Sci 11:1197. 10.3389/fpls.2020.01197 32849742 10.3389/fpls.2020.01197 PMC7426495

[CR4] Ata-Ul-Karim ST, Begum H, Lopena V, Borromeo T, Virk P, Hernandez JE, Gregorio GB, Collard BCY, Kato Y (2022) Genotypic variation of yield-related traits in an irrigated rice breeding program for tropical Asia. Crop Environ 1(3):173–181. 10.1016/j.crope.2022.08.004

[CR5] Babu PM, Neeraja CN, Rathod S, Suman K, Uttam GA, Chakravartty N, Lachagari VBR, Chaitanya U, Rao LVS, Voleti SR (2020) Stable SNP allele associations with high grain zinc content in polished rice (*Oryza sativa* L.) identified based on ddRAD sequencing. Front Genet 11(August):1–18. 10.3389/fgene.2020.00763 32849786 10.3389/fgene.2020.00763 PMC7432318

[CR6] Bhuvaneswari S, Krishnan SG, Ellur RK, Vinod KK, Bollinedi H, Bhowmick PK, Bansal VP, Nagarajan M, Singh AK (2020) Discovery of a novel induced polymorphism in SD1 gene governing semi-dwarfism in rice and development of a functional marker for marker-assisted selection. Plants. 10.3390/plants9091198 32937792 10.3390/plants9091198 PMC7570060

[CR7] Bouis HE, Saltzman A (2017) Improving nutrition through biofortification: a review of evidence from HarvestPlus, 2003 through 2016. Global Food Secur 12:49–58. 10.1016/j.gfs.2017.01.009 10.1016/j.gfs.2017.01.009 PMC5439484 28580239

[CR8] Bradbury PJ, Zhang Z, Kroon DE, Casstevens TM, Ramdoss Y, Buckler ES (2007) TASSEL: software for association mapping of complex traits in diverse samples. Bioinformatics 23(19):2633–2635. 10.1093/bioinformatics/btm308 17586829 10.1093/bioinformatics/btm308

[CR9] Calayugan MIC, Formantes AK, Amparado A, Descalsota-Empleo GIL, Nha CT, Inabangan-Asilo MA, Swe ZM, Hernandez JE, Borromeo TH, Lalusin AG, Mendioro MS, Diaz MGQ, Viña CBD, Reinke R, Swamy BPM (2020) Genetic analysis of agronomic traits and grain iron and zinc concentrations in a doubled haploid population of rice (*Oryza sativa* L.). Sci Rep. 10.1038/s41598-020-59184-z 32042046 10.1038/s41598-020-59184-z PMC7010768

[CR10] Calayugan MIC, Swamy BPM, Nha CT, Palanog AD, Biswas PS, Descalsota-Empleo GIL, Min YMM, Inabangan-Asilo MA (2021) Zinc-biofortified rice: a sustainable food-based product for fighting zinc malnutrition. Rice improvement. Springer, pp 449–470. 10.1007/978-3-030-66530-2_13

[CR11] Cullis BR, Smith AB, Coombes NE (2006) On the design of early generation variety trials with correlated data. J Agric Biol Environ Stat 11(4):381. 10.1198/108571106X154443

[CR12] De Leon TB, Linscombe S, Subudhi PK (2016) Molecular dissection of seedling salinity tolerance in rice (*Oryza sativa* L.) using a high-density GBS-based SNP linkage map. Rice (New York, NY) 9(1):52. 10.1186/s12284-016-0125-2 10.1186/s12284-016-0125-2 PMC5045836 27696287

[CR13] Descalsota GIL, Swamy BPM, Zaw H, Inabangan-Asilo MA, Amparado A, Mauleon R, Chadha-Mohanty P, Arocena EC, Raghavan C, Leung H, Hernandez JE, Lalusin AB, Mendioro MS, Diaz MGQ, Reinke R (2018) Genome-wide association mapping in a rice magic plus population detects qtls and genes useful for biofortification. Front Plant Sci 9:1–20. 10.3389/fpls.2018.01347 30294335 10.3389/fpls.2018.01347 PMC6158342

[CR14] Descalsota GIL, Amparado A, Inabangan-Asilo MA, Tesoro F, Stangoulis J, Reinke R, Swamy BPM (2019a) Genetic mapping of QTL for agronomic traits and grain mineral elements in rice. The Crop Journal 7(4):560–572. 10.1016/j.cj.2019.03.002

[CR15] Descalsota GIL, Noraziyah AAS, Navea IP, Chung C, Dwiyanti MS, Labios RJD, Ikmal AM, Juanillas VM, Inabangan-Asilo MA, Amparado A, Reinke R, Cruz CMV, Chin JH, Swamy BPM (2019) Genetic dissection of grain nutritional traits and leaf blight resistance in rice. Genes. 10.3390/genes10010030 10.3390/genes10010030 PMC6356647 30626141

[CR16] Dhurai SY, Mohan Reddy D, Ravi S (2016) Correlation and path analysis for yield and quality characters in rice (*Oryza sativa* L.). Rice Genomics Genet. 10.5376/rgg.2016.07.0004

[CR17] Farooq DM, Alamri AF, Alwhahabi BK, Metwally AM, Kareem KA (2020) The status of zinc in type 2 diabetic patients and its association with glycemic control. J Fam Commun Med 27(1):29–36. 10.4103/jfcm.JFCM_113_19 10.4103/jfcm.JFCM_113_19 PMC6984028 32030076

[CR18] Furuta T, Ashikari M, Jena KK, Doi K, Reuscher S (2017) Adapting genotyping-by-sequencing for rice F_2_ populations. G3 Genes|Genomes|Genetics 7(3):881–893. 10.1534/g3.116.038190 28082325 10.1534/g3.116.038190 PMC5345719

[CR19] Gangashetty P, Salimath PM, Hanamaratti NG (2013) Association analysis in genetically diverse non-basmati local aromatic genotypes of rice (*Oryza sativa* L). Mol Plant Breed 4(4):31–37

[CR20] Global Nutrition Report (2021) The state of global nutrition. 55

[CR21] Guild GE, Paltridge NG, Andersson MS, Stangoulis JCR (2017) An energy-dispersive X-ray fluorescence method for analysing Fe and Zn in common bean, maize and cowpea biofortification programs. Plant Soil 419(1–2):457–466. 10.1007/s11104-017-3352-4 32968327 10.1007/s11104-017-3352-4 PMC7473100

[CR22] Huang S, Yamaji N, Feng MJ (2022) Zinc transport in rice: how to balance optimal plant requirements and human nutrition. J Exp Bot 73(6):1800–1808. 10.1093/jxb/erab478 34727182 10.1093/jxb/erab478

[CR23] Hussein M, Fathy W, Hassan A, Elkareem RA, Marzouk S, Kamal YS (2021) Zinc deficiency correlates with severity of diabetic polyneuropathy. Brain Behav 11(10):e2349–e2349. 10.1002/brb3.2349 34521153 10.1002/brb3.2349 PMC8553312

[CR24] Inabangan-Asilo MA, Descalsota GIL, Nha CT, Calayugan MIC, Panalog AD, Sue ZM, Arocena EC, Amparado A, Tesoro F, Marfori-Wazarea CM (2019) Exploring Aus germplasm for breeding high zinc rice varieties. Philipp J Crop Sci (Philipp) 44:124

[CR25] IRRI. (2013). Standard Evaluation System for Rice. International Rice Research Instiitute, June, 55. http://www.clrri.org/ver2/uploads/SES_5th_edition.pdf

[CR26] IRRI (2014) Plant breeding tools (PB Tools), Version: 1.4, International Rice Research Institute, Los Banos

[CR27] Islam MZ, Arifuzzaman M, Banik S, Hossain MA, Ferdous J, Khalequzzaman M, Pittendrigh BR, Tomita M, Ali MP (2020) Mapping QTLs underpin nutrition components in aromatic rice germplasm. PLoS ONE 15(6):e0234395. 10.1371/journal.pone.0234395 32525930 10.1371/journal.pone.0234395 PMC7289389

[CR28] Jiang S, Wu JG, Nguyen T, Feng Y, Yang X, Shi C (2008) Genotypic variation of mineral elements contents in rice (*Oryza sativa* L.). Eur Food Res Technol 228:115–122. 10.1007/s00217-008-0914-y

[CR29] Jin T, Chen J, Zhu L, Zhao Y, Guo J, Huang Y (2015) Comparative mapping combined with homology-based cloning of the rice genome reveals candidate genes for grain zinc and iron concentration in maize. BMC Genet 16(1):17. 10.1186/s12863-015-0176-1 25888360 10.1186/s12863-015-0176-1 PMC4377022

[CR30] Johnson AAT, Kyriacou B, Callahan DL, Carruthers L, Stangoulis J, Lombi E, Tester M (2011) Constitutive overexpression of the *OsNAS* gene family reveals single-gene strategies for effective iron-and zinc-biofortification of rice endosperm. PLoS ONE 6(9):e24476 21915334 10.1371/journal.pone.0024476 PMC3167849

[CR31] Joshi G, Soe YP, Palanog A, Hore TK, Nha CT, Calayugan MI, Inabangan-Asilo MA, Amparado A, Pandey ID, Cruz PCS, Hernandez JE, Swamy BPM (2023) Meta-QTL s and haplotypes for efficient zinc biofortification of rice. Plant Genome. 10.1002/tpg2.20315 36896580 10.1002/tpg2.20315 PMC12807186

[CR32] Kader MA, Biswas PS, Aditya TL, Anisuzzaman M, Hore TK, Haq ME (2020) Zinc enriched high yielding rice variety BRRI dhan84 for dry season rice growing areas of Bangladesh. Asian Plant Res J. 10.9734/aprj/2020/v6i130117

[CR33] Kader MA, Shalahuddin AKM, Hore TK, Majumder RR, Haq ME, Fatema K, Biswas PS, Iftekharuddaula KM (2021) BRRI Dhan100: a zinc enriched rice variety suitable for irrigated ecosystem in Bangladesh. Asian Plant Res J 8(1):1–8. 10.9734/aprj/2021/v8i130164

[CR34] Kaler AS, Gillman JD, Beissinger T, Purcell LC (2020) Comparing different statistical models and multiple testing corrections for association mapping in soybean and maize. Front Plant Sci 10:01794. 10.3389/fpls.2019.01794 10.3389/fpls.2019.01794 PMC7052329 32158452

[CR35] Kosambi DD (1944) The estimation of map distances from recombinant values. Ann Eugen 12:172–175

[CR36] Kumar A, Sandhu N, Dixit S, Yadav S, Swamy BPM, Shamsudin NAA (2018) Marker-assisted selection strategy to pyramid two or more QTLs for quantitative trait-grain yield under drought. Rice 11(1):35. 10.1186/s12284-018-0227-0 29845495 10.1186/s12284-018-0227-0 PMC5975061

[CR37] Lekklar C, Pongpanich M, Suriya-arunroj D, Chinpongpanich A, Tsai H, Comai L, Chadchawan S, Buaboocha T (2019) Genome-wide association study for salinity tolerance at the flowering stage in a panel of rice accessions from Thailand. BMC Genomics 20(1):76. 10.1186/s12864-018-5317-2 30669971 10.1186/s12864-018-5317-2 PMC6343365

[CR38] Li H, Ye G, Wang J (2007) A modified algorithm for the improvement of composite interval mapping. Genetics 175(1):361–374. 10.1534/genetics.106.066811 17110476 10.1534/genetics.106.066811 PMC1775001

[CR39] Li H, Ribaut JM, Li Z, Wang J (2008) Inclusive composite interval mapping (ICIM) for digenic epistasis of quantitative traits in biparental populations. Theor Appl Genet 116(2):243–260. 10.1007/s00122-007-0663-5 17985112 10.1007/s00122-007-0663-5

[CR40] Lu K, Li L, Zheng X, Zhang Z, Mou T, Hu Z (2009) Quantitative trait loci controlling Cu, Ca, Zn, Mn and Fe content in rice grains. J Genet 87:305–310. 10.1007/s12041-008-0049-8 10.1007/s12041-008-0049-8 19147920

[CR41] Lyra DH, de Freitas ML, Galli G, Alves FC, Granato ÍSC, Fritsche-Neto R (2017) Multi-trait genomic prediction for nitrogen response indices in tropical maize hybrids. Mol Breed 37(6):80. 10.1007/s11032-017-0681-1

[CR42] Malav AK, Indu CKS (2016) Gene pyramiding: an overview. Int J Curr Res Biosci Plant Biol 3:22–28

[CR43] Meng L, Li H, Zhang L, Wang J (2015) QTL IciMapping: integrated software for genetic linkage map construction and quantitative trait locus mapping in biparental populations. Crop J 3(3):269–283. 10.1016/j.cj.2015.01.001

[CR44] Mohiuddin S, Haque M, Haque MM, Islam T, Biswas P (2020) Genetic analysis reveals a major effect QTL associated with high grain zinc content in rice (*Oryza sativa* L.). Plant Breed Biotechnol 8:327–340. 10.9787/PBB.2020.8.4.327

[CR45] Morales KY, Singh N, Perez FA, Ignacio JC, Thapa R, Arbelaez JD, Tabien RE, Famoso A, Wang DR, Septiningsih EM, Shi Y, Kretzschmar T, McCouch SR, Thomson MJ (2020) An improved 7K SNP array, the C7AIR, provides a wealth of validated SNP markers for rice breeding and genetics studies. PLoS ONE 15(5):1–14. 10.1371/journal.pone.0232479 10.1371/journal.pone.0232479 PMC7224494 32407369

[CR46] Mu S, Yamaji N, Sasaki A, Luo L, Du B, Che J, Shi H, Zhao H, Huang S, Deng F, Shen Z, Lou GM, Zheng L, Ma JF (2021) A transporter for delivering zinc to the developing tiller bud and panicle in rice. Plant J for Cell Mol Biol 105(3):786–799. 10.1111/tpj.15073 10.1111/tpj.15073 33169459

[CR47] Murray MG, Thompson WF (1980) Rapid isolation of high molecular weight plant DNA. Nucleic Acids Res 8(19):4321–4326. 10.1093/nar/8.19.4321 7433111 10.1093/nar/8.19.4321 PMC324241

[CR48] Muvunyi BP, Zou W, Zhan J, He S, Ye G (2022) Multi-trait genomic prediction models enhance the predictive ability of grain trace elements in rice. Front Genet. 10.3389/fgene.2022.883853 35812754 10.3389/fgene.2022.883853 PMC9257107

[CR49] Neeraja CN, Babu VR, Ram S, Hossain F, Hariprasanna K, Rajpurohit BS, Prabhakar, Longvah T, Prasad KS, Sandhu JS (2017) Biofortification in cereals: progress and prospects. Curr Sci 113:1050–1057

[CR50] Norton GJ, Deacon CM, Xiong L, Huang S, Meharg AA, Price AH (2010) Genetic mapping of the rice ionome in leaves and grain: identification of QTLs for 17 elements including arsenic, cadmium, iron and selenium. Plant Soil 329(1):139–153. 10.1007/s11104-009-0141-8

[CR51] Nuthalapati C, Joshi P, Mittra B, Pingali P (2022) Nutrition-sensitive food systems and biofortified crops. Agric Econ Res Rev 35:123–132. 10.5958/0974-0279.2022.00011.8

[CR52] Palanog AD, Calayugan MIC, Descalsota-Empleo GIL, Amparado A, Inabangan-Asilo MA, Arocena EC, Cruz PC Sta., Borromeo TH, Lalusin A, Hernandez JE, Acuin C, Reinke R, Swamy BPM (2019) Zinc and iron nutrition status in the Philippines population and local soils. Front Nutr 6:81. 10.3389/fnut.2019.00081 31231657 10.3389/fnut.2019.00081 PMC6568233

[CR53] Palanog AD, Nha CT, Descalsota-Empleo GIL, Calayugan MIC, Swe ZM, Amparado A, Inabangan-Asilo MA, Hernandez JE, Cruz PC Sta., Borromeo TH, Lalusin AG, Mauleon R, McNally KL, Swamy BPM (2023) Molecular dissection of connected rice populations revealed important genomic regions for agronomic and biofortification traits. Front Plant Sci 14:1157507. 10.3389/fpls.2023.1157507 37035067 10.3389/fpls.2023.1157507 PMC10073715

[CR54] Peramaiyan P, Craufurd P, Kumar V, Seelan LP, Mcdonald AJ, Balwinder-Singh, Kishore A, Singh S (2022) Agronomic biofortification of zinc in rice for diminishing malnutrition in South Asia. Sustainability 14(13):su14137747. 10.3390/su14137747

[CR55] Pešek J, Baker RJ (1969) Desired improvement in relation to selection indices. Can J Plant Sci 49(6):803–804. 10.4141/cjps69-137

[CR56] Piepho HP, Möhring J (2007) Computing heritability and selection response from unbalanced plant breeding trials. Genetics 177(3):1881–1888. 10.1534/genetics.107.074229 18039886 10.1534/genetics.107.074229 PMC2147938

[CR57] Pradhan SK, Pandit E, Pawar S, Naveenkumar R, Barik SR, Mohanty SP, Nayak DK, Ghritlahre SK, Sanjiba Rao D, Reddy JN, Patnaik SSC (2020) Linkage disequilibrium mapping for grain Fe and Zn enhancing QTLs useful for nutrient dense rice breeding. BMC Plant Biol 20(1):57. 10.1186/s12870-020-2262-4 32019504 10.1186/s12870-020-2262-4 PMC7001215

[CR58] Prasad AS (2004) Zinc deficiency: its characterization and treatment. Met Ions Biol Syst 41:103–138 15206115

[CR59] R core Software (2012) A language and environment for statistical computing (R Foundation for Statistical Computing, Vienna, 2012)

[CR60] Rahman MA, Bimpong IK, Bizimana JB, Pascual ED, Arceta M, Swamy BPM, Dia F, Rahman MS, Singh RK (2017) Mapping QTLs using a novel source of salinity tolerance from Hasawi and their interaction with environments in rice. Rice 10(1):47. 10.1186/s12284-017-0186-x 29098463 10.1186/s12284-017-0186-x PMC5668218

[CR61] Rakotondramanana M, Tanaka R, Pariasca-Tanaka J, Stangoulis J, Grenier C, Wissuwa M (2022) Genomic prediction of zinc-biofortification potential in rice gene bank accessions. Theor Appl Genet 135(7):2265–2278. 10.1007/s00122-022-04110-2 35618915 10.1007/s00122-022-04110-2 PMC9271118

[CR62] Rana MM, Takamatsu T, Baslam M, Kaneko K, Itoh K, Harada N, Sugiyama T, Ohnishi T, Kinoshita T, Takagi H, Mitsui T (2019) Salt tolerance improvement in rice through efficient SNP marker-assisted selection coupled with speed-breeding. Int J Mol Sci. 10.3390/ijms20102585 31130712 10.3390/ijms20102585 PMC6567206

[CR63] Raza Q, Riaz A, Sabar M, Atif RM, Bashir K (2019) Meta-analysis of grain iron and zinc associated QTLs identified hotspot chromosomal regions and positional candidate genes for breeding biofortified rice. Plant Sci 288:110214. 10.1016/j.plantsci.2019.110214 31521222 10.1016/j.plantsci.2019.110214

[CR64] Ryu MS, Aydemir TB (2020) Chapter 23: Zinc. In: Marriott BP, Birt DF, Stallings VA, Yates AABT (eds) Present knowledge in nutrition, 11th edn. Academic Press, London, pp 393–408. 10.1016/B978-0-323-66162-1.00023-8

[CR65] Sasaki T, Project IRGS (2005) The map-based sequence of the rice genome. Nature 436(7052):793–800. 10.1038/nature03895 16100779 10.1038/nature03895

[CR66] Sasaki A, Yamaji N, Ma JF (2014) Overexpression of *OsHMA3* enhances Cd tolerance and expression of Zn transporter genes in rice. J Exp Bot 65(20):6013–6021 25151617 10.1093/jxb/eru340 PMC4203134

[CR67] Sasaki A, Yamaji N, Mitani-Ueno N, Kashino M, Ma JF (2015) A node-localized transporter *OsZIP3* is responsible for the preferential distribution of Zn to developing tissues in rice. Plant J Cell Mol Biol 84(2):374–384. 10.1111/tpj.13005 10.1111/tpj.13005 26332571

[CR68] Satoh-Nagasawa N, Mori M, Nakazawa N, Kawamoto T, Nagato Y, Sakurai K, Takahashi H, Watanabe A, Akagi H (2012) Mutations in rice (*Oryza sativa*) heavy metal ATPase 2 (*OsHMA2*) restrict the translocation of zinc and cadmium. Plant Cell Physiol 53(1):213–224. 10.1093/pcp/pcr166 22123790 10.1093/pcp/pcr166

[CR69] Semba RD, Askari S, Gibson S, Bloem MW, Kraemer K (2022) The potential impact of climate change on the micronutrient-rich food supply. Adv Nutr (Bethesda, Md) 13(1):80–100. 10.1093/advances/nmab104 10.1093/advances/nmab104 PMC8803495 34607354

[CR70] Shahzad Z, Rouached H, Rakha A (2014) Combating mineral malnutrition through iron and zinc biofortification of cereals. Compr Rev Food Sci Food Saf 13(3):329–346 33412655 10.1111/1541-4337.12063

[CR71] Singh V, Singh AK, Mohapatra TS, Gopala Krishnan S, Ellur RK (2018) Pusa Basmati 1121: a rice variety with exceptional kernel elongation and volume expansion after cooking. Rice 11(1):19. 10.1186/s12284-018-0213-6 29629488 10.1186/s12284-018-0213-6 PMC5890003

[CR72] Singhal T, Satyavathi CT, Singh SP, Kumar A, Sankar SM, Bhardwaj C, Mallik M, Bhat J, Anuradha N, Singh N (2021) Multi-environment quantitative trait loci mapping for grain iron and zinc content using bi-parental recombinant inbred line mapping population in pearl millet. Front Plant Sci. 10.3389/fpls.2021.659789 34093617 10.3389/fpls.2021.659789 PMC8169987

[CR73] Suman K, Neeraja CN, Madhubabu P, Rathod S, Bej S, Jadhav KP, Kumar JA, Chaitanya U, Pawar SC, Rani SH, Subbarao LV, Voleti SR (2021) Identification of promising RILs for high grain zinc through genotype × environment analysis and stable grain zinc QTL using SSRs and SNPs in rice (*Oryza sativa* L.). Front Plant Sci 12:587482. 10.3389/fpls.2021.587482 33679823 10.3389/fpls.2021.587482 PMC7930840

[CR74] Swamy BPM, Rahman MA, Inabangan-Asilo MA, Amparado A, Manito C, Chadha-Mohanty P, Reinke R, Slamet-Loedin IH (2016) Advances in breeding for high grain Zinc in Rice. Rice 9(1):49. 10.1186/s12284-016-0122-5 27671163 10.1186/s12284-016-0122-5 PMC5037106

[CR75] Swamy BPM, Descalsota GIL, Nha CT, Amparado A, Inabangan-Asilo MA, Manito C, Tesoro F, Reinke R (2018) Identification of genomic regions associated with agronomic and biofortification traits in DH populations of rice. PloS ONE. 10.1371/journal.pone.0201756 30096168 10.1371/journal.pone.0201756 PMC6086416

[CR76] Swamy BPM, Marundan S, Samia M, Ordonio RL, Rebong DB, Miranda R, Alibuyog A, Rebong AT, Tabil MA, Suralta RR, Alfonso AA, Biswas PS, Kader MA, Reinke R, Boncodin R, MacKenzie DJ (2021) Development and characterization of GR2E Golden rice introgression lines. Sci Rep 11(1):1–12. 10.1038/s41598-021-82001-0 33510272 10.1038/s41598-021-82001-0 PMC7843986

[CR77] Symonds VV, Godoy AV, Alconada T, Botto JF, Juenger TE, Casal JJ, Lloyd AM (2005) Mapping quantitative trait loci in multiple populations of *Arabidopsis thaliana* identifies natural allelic variation for trichome density. Genetics 169(3):1649–1658. 10.1534/genetics.104.031948 15654092 10.1534/genetics.104.031948 PMC1449524

[CR78] Takahashi R, Bashir K, Ishimaru Y, Nishizawa NK, Nakanishi H (2012a) The role of heavy-metal ATPases, HMAs, in zinc and cadmium transport in rice. Plant Signal Behav 7(12):1605–1607. 10.4161/psb.22454 23072989 10.4161/psb.22454 PMC3578901

[CR79] Takahashi R, Ishimaru Y, Shimo H, Ogo Y, Senoura T, Nishizawa NK, Nakanishi H (2012b) The *OsHMA2* transporter is involved in root-to-shoot translocation of Zn and Cd in rice. Plant Cell Environ 35(11):1948–1957. 10.1111/j.1365-3040.2012.02527.x 22548273 10.1111/j.1365-3040.2012.02527.x

[CR80] Tan L, Zhu Y, Fan T, Peng C, Wang J, Sun L, Chen C (2019) *OsZIP7* functions in xylem loading in roots and inter-vascular transfer in nodes to deliver Zn/Cd to grain in rice. Biochem Biophys Res Commun 512(1):112–118. 10.1016/j.bbrc.2019.03.024 30871778 10.1016/j.bbrc.2019.03.024

[CR81] Thompson MW (2022) Regulation of zinc-dependent enzymes by metal carrier proteins. Biometals 35(2):187–213. 10.1007/s10534-022-00373-w 35192096 10.1007/s10534-022-00373-w PMC8862405

[CR82] Uttam GA, Suman K, Jaldhani V, Babu PM, Rao DS, Sundaram RM, Neeraja CN (2023) Identification of genomic regions associated with high grain Zn content in polished rice using genotyping-by-sequencing (GBS). Plants 12(1):12010144. 10.3390/plants12010144 10.3390/plants12010144 PMC9824299 36616273

[CR83] Vikram P, Swamy BPM, Dixit S, Singh R, Singh B, Miro B, Kohli A, Henry A, Singh NK, Kumar A (2015) Drought susceptibility of modern rice varieties: an effect of linkage of drought tolerance with undesirable traits. Sci Rep 5(1):14799. 10.1038/srep14799 26458744 10.1038/srep14799 PMC4602206

[CR84] Vogel-González M, Talló-Parra M, Herrera-Fernández V, Pérez-Vilaró G, Chillón M, Nogués X, Gómez-Zorrilla S, López-Montesinos I, Arnau-Barrés I, Sorli-Redó ML, Horcajada JP, García-Giralt N, Pascual J, Díez J, Vicente R, Güerri-Fernández R (2021) Low zinc levels at admission associates with poor clinical outcomes in SARS-CoV-2 infection. Nutrients 13(2):562. 10.3390/nu13020562 33572045 10.3390/nu13020562 PMC7914437

[CR85] Wani SH, Gaikwad K, Razzaq A, Samantara K, Kumar M, Govindan V (2022) Improving zinc and iron biofortification in wheat through genomics approaches. Mol Biol Rep 49(8):8007–8023. 10.1007/s11033-022-07326-z 35661970 10.1007/s11033-022-07326-z PMC9165711

[CR86] Wen Y, Fang Y, Hu P, Tan Y, Wang Y, Hou L, Deng X, Wu H, Zhu L, Zhu L, Chen G, Zeng D, Guo L, Zhang G, Gao Z, Dong G, Ren D, Shen L, Zhang Q, Hu J (2020) Construction of a high-density genetic map based on SLAF markers and QTL analysis of leaf size in rice. Front Plant Sci. 10.3389/fpls.2020.01143 32849702 10.3389/fpls.2020.01143 PMC7411225

[CR87] Wessells KR, Brown KH (2012) Estimating the global prevalence of zinc deficiency: results based on zinc availability in national food supplies and the prevalence of stunting. PLoS ONE 7(11):e50568. 10.1371/journal.pone.0050568 23209782 10.1371/journal.pone.0050568 PMC3510072

[CR88] Wissuwa M, Ismail AM, Graham RD (2008) Rice grain zinc concentrations as affected by genotype, native soil-zinc availability, and zinc fertilization. Plant Soil 306(1):37–48

[CR89] Xu Y, Li P, Yang Z, Xu C (2017) Genetic mapping of quantitative trait loci in crops. Crop J 5(2):175–184. 10.1016/j.cj.2016.06.003

[CR90] Yamaji N, Xia J, Mitani-Ueno N, Yokosho K, Feng MJ (2013) Preferential delivery of zinc to developing tissues in rice is mediated by P-type heavy metal ATPase *OsHMA2*. Plant Physiol 162(2):927–939 23575418 10.1104/pp.113.216564 PMC3668081

[CR91] Yang Z, Jin L, Zhu H, Wang S, Zhang G, Liu G (2018) Analysis of epistasis among QTLs on heading date based on single segment substitution lines in rice. Sci Rep 8(1):3059. 10.1038/s41598-018-20690-w 29449579 10.1038/s41598-018-20690-w PMC5814450

[CR92] Yassue RM, Sabadin F, Galli G, Alves FC, Fritsche-Neto R (2021) CV-α: designing validations sets to increase the precision and enable multiple comparison tests in genomic prediction. Euphytica 217(6):106. 10.1007/s10681-021-02831-x

[CR93] Yu Y, Hu X, Zhu Y, Mao D (2020) Re-evaluation of the rice ‘Green Revolution’ gene: the weak allele SD1-EQ from japonica rice may be beneficial for super indica rice breeding in the post-Green Revolution era. Mol Breeding 40(9):84. 10.1007/s11032-020-01164-2

[CR94] Zaghum MJ, Ali K, Teng S (2022) Integrated genetic and omics approaches for the regulation of nutritional activities in rice (*Oryza sativa* L.). Agriculture 12(11):1757. 10.3390/agriculture12111757

[CR95] Zaw H, Raghavan C, Pocsedio A, Swamy BPM, Jubay ML, Singh RK, Bonifacio J, Mauleon R, Hernandez JE, Mendioro MS, Gregorio GB, Leung H (2019) Exploring genetic architecture of grain yield and quality traits in a 16-way indica by japonica rice MAGIC global population. Sci Rep 9(1):1–11. 10.1038/s41598-019-55357-7 31862941 10.1038/s41598-019-55357-7 PMC6925145

[CR96] Zhang Z, Ersoz E, Lai CQ, Todhunter RJ, Tiwari HK, Gore MA, Bradbury PJ, Yu J, Arnett DK, Ordovas JM, Buckler ES (2010) Mixed linear model approach adapted for genome-wide association studies. Nat Genet 42(4):355–360. 10.1038/ng.546 20208535 10.1038/ng.546 PMC2931336

[CR97] Zhu M, Zhao S (2007) Candidate gene identification approach: progress and challenges. Int J Biol Sci 3(7):420–427. 10.7150/ijbs.3.420 17998950 10.7150/ijbs.3.420 PMC2043166

