# Supplementary material for: Genomic prediction and QTL analysis for grain Zn content and yield in Aus-derived rice populations
Source: J Plant Biochem Biotechnol. 2024 May 9;33(2):216–36. doi: 10.1007/s13562-024-00886-0 (PMC12037680; doi:10.1007/s13562-024-00886-0)
Supplement: Supplementary file 1 — Supplementary file1 (DOCX 249 KB) [file 13562_2024_886_MOESM1_ESM.docx]

**Table S1: Details PCA for agronomic and grain Zn traits in four mapping populations**

| **Variable** | **P1** | | **P2** | | **P3** | | **P4** | |
| --- | --- | --- | --- | --- | --- | --- | --- | --- |
|  | **PC1** | **PC2** | **PC1** | **PC2** | **PC1** | **PC2** | **PC1** | **PC2** |
| Eigenvalue | 2.36 | 1.74 | 2.01 | 1.67 | 2.46 | 1.77 | 2.39 | 1.66 |
| Variability (%) | 29.46 | 21.72 | 25.17 | 20.88 | 30.74 | 22.17 | 29.86 | 20.72 |
| Cumulative (%) | 29.46 | 51.18 | 25.17 | 46.05 | 30.74 | 52.91 | 29.86 | 50.58 |
| DF (days) | -0.15 | 0.34 | -0.36 | -0.37 | 0.45 | -0.32 | 0.28 | 0.56 |
| PH (cm) | -0.75 | 0.14 | -0.59 | 0.24 | 0.58 | 0.09 | 0.63 | 0.07 |
| PL (cm) | -0.68 | 0.29 | -0.59 | -0.05 | 0.71 | -0.06 | 0.67 | 0.23 |
| PN | 0.66 | 0.63 | 0.50 | -0.66 | -0.63 | -0.66 | -0.72 | 0.58 |
| TN | 0.67 | 0.65 | 0.46 | -0.67 | -0.61 | -0.68 | -0.68 | 0.64 |
| YLD(t/ha) | -0.52 | 0.49 | -0.55 | -0.32 | 0.57 | -0.52 | 0.38 | 0.43 |
| Zn(ppm) | 0.24 | -0.57 | 0.55 | 0.47 | -0.42 | 0.56 | -0.48 | -0.38 |

**Table S2: Linkage map information**

| **Chr** | **P1** | | **P2** | | **P3** | | **P4** | |
| --- | --- | --- | --- | --- | --- | --- | --- | --- |
|  | **SNPs** | **ML (cM)** | **SNPs** | **ML (cM)** | **SNPs** | **ML (cM)** | **SNPs** | **ML (cM)** |
| **Chr1** | 75 | 178.87 | 36 | 228.26 | 66 | 244.52 | 97 | 99.29 |
| **Chr2** | 99 | 274.75 | 35 | 86.8 | 73 | 201.95 | 95 | 260.47 |
| **Chr3** | 92 | 233.51 | 11 | 185.87 | 17 | 130.6 | 81 | 203.14 |
| **Chr4** | 97 | 242.67 | 28 | 186.93 | 30 | 147.55 | 71 | 175.18 |
| **Chr5** | 59 | 131.36 | 11 | 154.49 | 21 | 146.2 | 72 | 90.21 |
| **Chr6** | 85 | 105.92 | 11 | 110.37 | 13 | 20.8 | 66 | 175.23 |
| **Chr7** | 67 | 92.01 | 6 | 5.98 | 18 | 145.51 | 31 | 71.78 |
| **Chr8** | 54 | 48.12 | 18 | 131.66 | 27 | 122.16 | 47 | 108.23 |
| **Chr9** | 59 | 67.01 | 21 | 105.06 | 49 | 126.44 | 48 | 187.18 |
| **Chr10** | 44 | 137.34 | 13 | 75.14 | 9 | 105.88 | 52 | 96.99 |
| **Chr11** | 67 | 58.12 | 14 | 182.15 | 31 | 182.71 | 71 | 154.08 |
| **Chr12** | 50 | 62.12 | 7 | 81.82 | 44 | 126.81 | 53 | 62.19 |
| **Total** | 848 | 1631.8 | 211 | 1534.53 | 398 | 1701.13 | 784 | 1683.97 |

**Chr: Chromosome, ML: Map length**

**
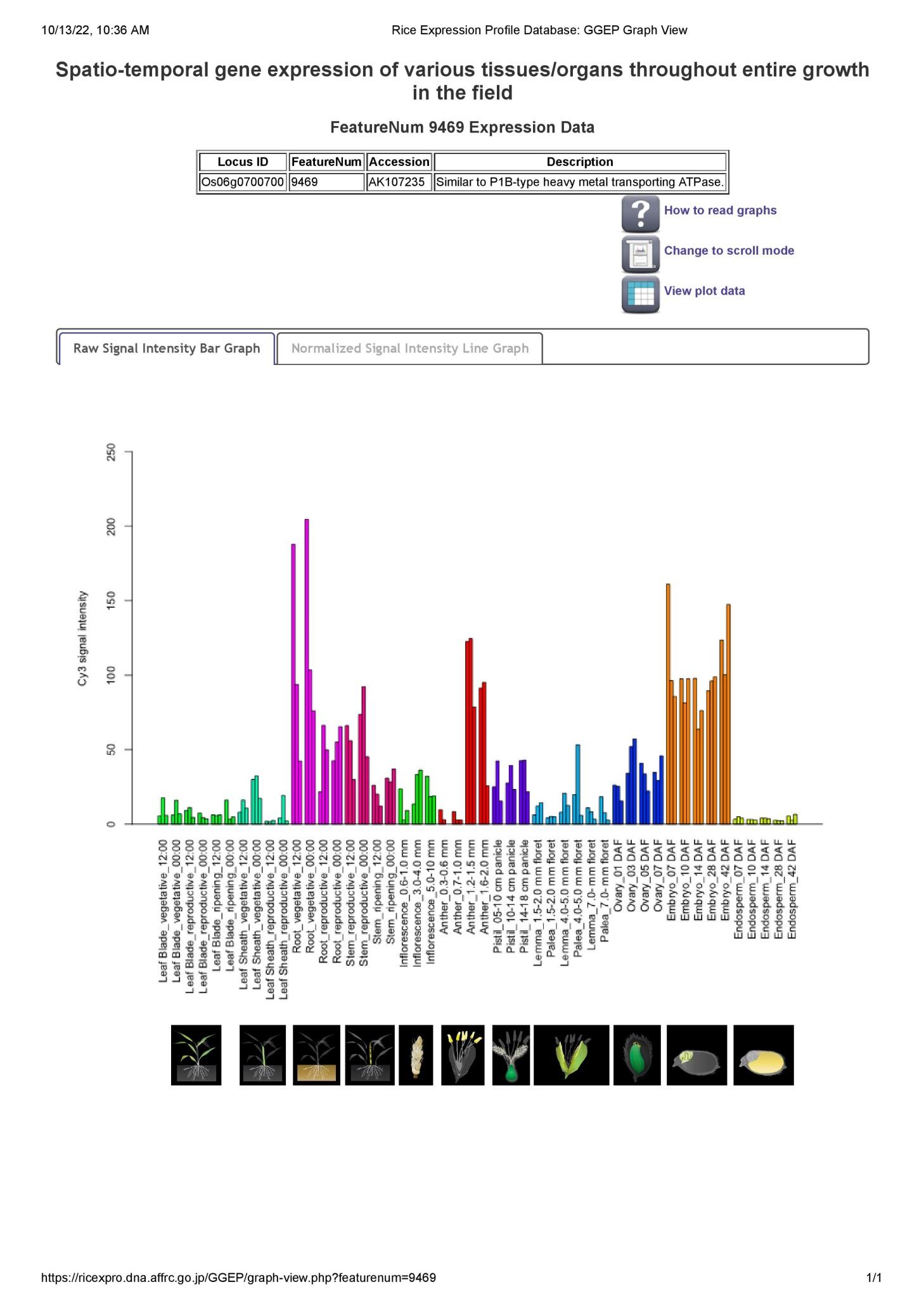
**

**Fig. S1: Expression pattern of *OsHMA2* in different parts of plant obtained using RiceXpro**
